# Two New Cytotoxic Sesquiterpene-Amino Acid Conjugates and a Coumarin-Glucoside from *Crossostephium chinense*

**DOI:** 10.3390/molecules28124696

**Published:** 2023-06-11

**Authors:** Zhichao Wang, Ben-Yeddy Abel Chitama, Keisuke Suganuma, Yoshi Yamano, Sachiko Sugimoto, Susumu Kawakami, Osamu Kaneko, Hideaki Otsuka, Katsuyoshi Matsunami

**Affiliations:** 1 Graduate School of Biomedical & Health Sciences, Hiroshima University, 1-2-3 Kasumi, Minami-ku, Hiroshima 734-8553, Japan; zhichao96425@gmail.com (Z.W.); yamano@hiroshima-u.ac.jp (Y.Y.); ssugimot@hiroshima-u.ac.jp (S.S.); 2 Department of Protozoology, Institute of Tropical Medicine (NEKKEN), Nagasaki University, 1-12-4 Sakamoto, Nagasaki 852-8523, Japan; benchitama@gmail.com (B.-Y.A.C.); okaneko@nagasaki-u.ac.jp (O.K.); 3 National Research Center for Protozoan Diseases, Obihiro University of Agriculture and Veterinary Medicine, Inada, Obihiro 080-8555, Japan; k.suganuma@obihiro.ac.jp; 4 Graduate School of Pharmacy, Yasuda Women’s University, Hiroshima 731-0153, Japan; kawakami@yasuda-u.ac.jp (S.K.); otsuka-h@yasuda-u.ac.jp (H.O.)

**Keywords:** *Crossostephium chinense*, sesquiterpene, A549, *Leishmania major*, *Plasmodium falciparum*, *Trypanosoma brucei*

## Abstract

The Asteraceae family is a promising source of bioactive compounds, such as the famous Asteraceae plants *Tanacetum cinerariifolium* (pyrethrin) and *Artemisia annua* (artemisinin). As a result of our series of phytochemical studies of the subtropical plants, two novel sesquiterpenes, named crossoseamines A and B in this study (**1** and **2**, respectively), one undescribed coumarin-glucoside (**3**), and eighteen known compounds (**4**–**21**) were isolated from the aerial part of *Crossostephium chinense* (Asteraceae). The structures of isolated compounds were elucidated by spectroscopic methods, including 1D and 2D NMR experiments (^1^H, ^13^C, DEPT, COSY, HSQC, HMBC, and NOESY), IR spectrum, circular dichroism spectrum (CD), and high-resolution electrospray ionization–mass spectrometry (HR-ESI–MS). All isolated compounds were evaluated for their cytotoxic activities against *Leishmania major*, *Plasmodium falciparum*, *Trypanosoma brucei* (*gambiense* and *rhodesiense*), and human lung cancer cell line A549 because of the high demand for the discovery of new drug leads to overcome the present side effects and emerging drug-resistant strains. As a result, the new compounds (**1** and **2**) showed significant activities against A549 (IC_50_, **1**: 3.3 ± 0.3; **2**: 12.3 ± 1.0 μg/mL), *L. major* (IC_50_, **1**: 6.9 ± 0.6; **2**: 24.9 ± 2.2 μg/mL), and *P. falciparum* (IC_50_, **1**: 12.1 ± 1.1; **2**: 15.6 ± 1.2 μg/mL).

## 1. Introduction

*Crossostephium chinense* (Asteraceae) is an evergreen shrub distributed in Japan, China, and other Asian countries. The shrub naturally grows on coastal raised coral reefs and can reach 10–40 cm in height. From November to December, the racemes are borne on the terminal branches and bloom tiny yellow flowers. *C. chinense* is widely planted as an ornamental shrub in China because of the high rate of survival for artificial cultivation [1]. The whole plants of *C. chinense* are traditionally used for the treatment of diabetes and arthritis in southward China [2]. The extract has been reported to have various activities, such as preventing the progression of restenosis [3], inhibiting differentiation, formation and bone resorptive abilities of osteoclasts [4], and larvicidal activity [5]. Flavonoids [6,7], coumarin [2,8], sesquiterpene [2], and triterpenoid [9] have previously been isolated from *C. chinense*; however, the phytochemical analysis is still insufficient, and a detailed evaluation of biological activities, especially for anticancer and antiparasitic activities, has yet to be conducted.

Cancer is a leading cause of death, especially in industrialized countries, and lung cancer causes the most deaths worldwide [10]. A549 is a cell line derived from human lung cancer and is frequently used in anticancer drug screening.

Leishmaniasis is a disease caused by protozoan parasites which are transmitted by the bite of infected phlebotomine sandflies. Some 1.5–2 million new cases occur annually [11], and emerging drug-resistant strains attract much attention for developing new chemical agents. *Leishmania major* is associated with cutaneous leishmaniasis, the most common form of leishmaniasis.

*Trypanosoma brucei gambiense* and *T. brucei rhodesiense* are the pathogens responsible for West and East African trypanosomiasis, also called sleeping sickness, respectively. Severe side effects and emerging resistant strains for the present clinical drugs have been reported [12,13].

*Plasmodium falciparum* is a unicellular protozoan parasite of humans and causes the most dangerous malaria, with a high risk of complication and mortality. According to the WHO malaria report of 2021, there were 247 million cases of malaria worldwide in 2021, resulting in an estimated 619,000 deaths. Artemisinin is a famous discovery from *Artemisia annua*, which belongs to the Asteraceae family and has saved millions of lives. However, artemisinin-resistant strains have been reported from southeastern Asia and Africa [14,15].

Our group focuses on the discovery of new bioactive compounds against these intractable diseases from natural resources. Asteraceae plants were one of the rich sources of bioactive compounds, such as pyrethrin from *Tanacetum cinerariifolium* and artemisinin from *A. anua*. Therefore, we are interested in an Asteraceae plant, *C. chinense*, to discover anticancer and antiparasitic constituents in this study.

The ethyl acetate soluble fraction from the aerial part of *C. chinense* was phytochemically studied, and all isolated compounds were investigated for their cytotoxic activities against human protozoan pathogens, *L. major*, *P. falciparum*, and *T. brucei* (*gambiense* and *rhodesiense*) and also against a human lung cancer cell line, A549. As a result, two novel sesquiterpene-amino acid conjugates (**1** and **2**) and one previously undescribed coumarin-glucoside (**3**) were discovered, of which two sesquiterpene-amino acid conjugates showed significant cytotoxic activity. Among the known compounds identified (**4**–**21**), the anti-*Trypanosoma* activity of **14** and anti-*Plasmodium* activity of compounds **1**, **2**, **11**, and **14** are reported for the first time. Although the anti-*Trypanosoma* activity of compounds **6**–**9** and **18** and the anti-*Plasmodium* activity of compounds **6**–**9**, **12**, **13**, **15**, and **18** have been previously reported, we tested these compounds using our experimental conditions to allow a direct comparison with the activities of **1** and **2** [16,17,18,19,20,21,22,23,24,25,26,27].

## 2. Results and Discussion

**Figure 1:**
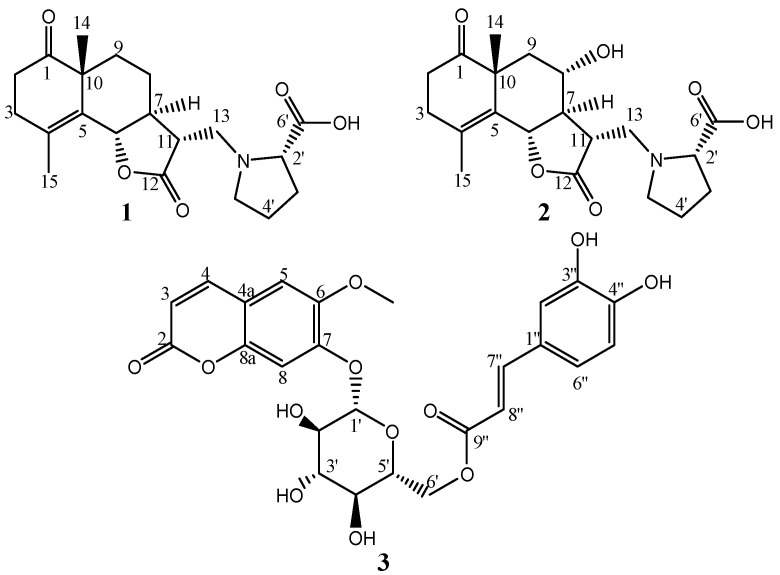
Structures of novel compounds (**1**–**3**) from *C. chinense*.

**Figure 2:**
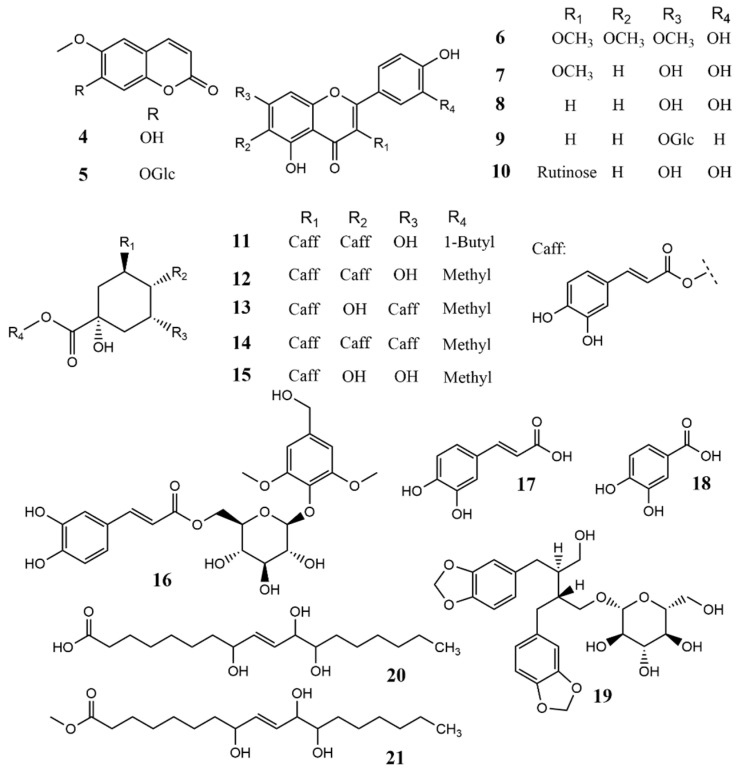
Structures of isolated known compounds (**4**–**21**) from *C. chinense*.

Extraction of *C. chinense* followed by liquid–liquid partitioning, column chromatography, and HPLC resulted in the isolation of two novel compounds (**1**, 0.00102% and **2**, 0.00175%) and a previously undescribed coumarin glucoside (**3**, 0.00125%) (Figure 1), together with the following 18 known compounds, i.e., which isolated and elucidated the structure previously, two coumarin derivatives (scopoletin (**4**, 0.0614%) [28] and scopolin (**5**, 0.00359%) [29,30]), five flavonoids and flavonoid glucosides (chrysosplenol D (**6**, 0.0354%) [31], 3-methylquercetin (**7**, 0.0166%) [32], luteolin (**8**, 0.0104%) [33], cosmosiine (**9**, 0.00168%) [34], quercetin-3-*O*-(6″-*O*-*α*-rhamnosyl)-*β*-glucoside (**10**, 0.00761%) [35]), seven caffeic acid derivatives (4,5-di-*O*-caffeoylquinic acid butyl ester (**11**, 0.0107%) [36], 4,5-di-*O*-caffeoylquinic acid methyl ester (**12**, 0.0136%) [37], 3,5-di-*O*-caffeoylquinic acid methyl ester (**13**, 0.0154%) [38], 3,4,5-tri-*O*-caffeoylqunic acid methyl ester (**14**, 0.00876%) [39], chlorogenic acid methyl ester (**15**, 0.0111%) [40], 2,6-dimethoxy-4-hydroxymethylphenol-1-*O*-(6-*O*-caffeoyl)-*β*-D-glucopyranoside (**16**, 0.00497%) [41], and caffeic acid (**17**, 0.00333%) [42]), 3,4-dihydroxy benzoic acid (**18**, 0.00639%) [43], one lignan (tetrecentronside B (**19**, 0.00477%) [44]), and two fatty acids (tianshic acid (**20**, 0.0241%) [45] and tianshic acid methyl ester (**21**, 0.00283%) [45]) (Figure 2). Known compounds were identified by comparison of their physicochemical data with those reported data. The structures of novel compounds were elucidated by 1D, 2D NMR, IR, CD, and HR-ESI-MS spectrometry and some chemical reactions.

### 2.1. Structure Determination of Crossoseamines A and B (**1** and **2**) and a Novel Coumarin Glucoside (**3**)

#### 2.1.1. Structure of Crossoseamine A (**1**)

**Figure 3:**
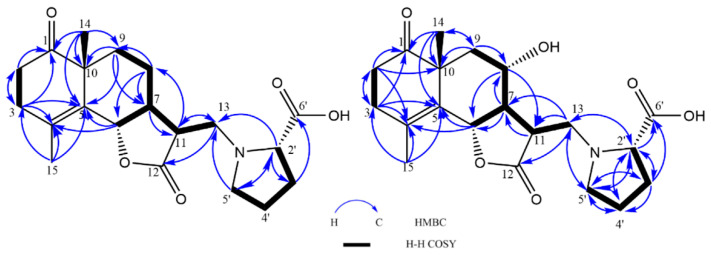
^1^H-^1^H COSY and HMBC correlations of **1** and **2**.

Crossoseamine A (**1**) was isolated as a colorless amorphous powder with a specific optical rotation (${\;[\alpha ]}_{D}^{27}-97,\;c\;0.2,\;\mathrm{MeOH}$) and was found to have a nitrogen function based on TLC examination using Dragendorff’s reagent. The molecular formula was determined as C_20_H_27_O_5_N by HR-ESI-MS data which revealed a protonated molecule [M + H]^+^ at *m*/*z* 362.1962 (calcd for C_20_H_28_O_5_N: 362.1961). The IR spectrum showed the absorption signals at 3411, 1762, and 1710 cm^−1^ due to hydroxy and carbonyl functions, of which 1762 cm^−1^ suggested a γ-lactone functional group. ^13^C NMR showed that of the eight indices of hydrogen deficiency, four were ascribed to one double bond (δ_C_ 125.2 and 130.0) and three carbonyl carbons (δ_C_ 212.9, 177.0, and 173.9) (Table 1). In addition, the spectra also displayed one oxymethine (δ_C_ 80.8), one aminomethine (δ_C_ 66.6), two methines (δ_C_ 48.5 and 44.2), one quaternary carbon (δ_C_ 48.4), and two methyl carbons (δ_C_ 19.4 and 23.0). The ^1^H-NMR spectrum (Table 1) showed a tertiary methyl, δ_H_ 1.25 (3H, s, H_3_-14), and a vinyl methyl, δ_H_ 1.86 (3H, br s, H_3_-15). The 1D NMR and HSQC spectrum displayed an oxymethine proton signal at δ_H_ 4.80 (1H, dquint-like, *J* = 11.2, 1.3 Hz, H-6) long-range coupled with the vinyl methyl group (H_3_-15) and one of the methylene protons of H_2_-3. The COSY correlations between H-5′ and methylene proton H-4′, H-4′ and H-3′, and H-3′ and methine proton H-2′ (Figure 3) and the ^13^C signals at δ_C_ 173.9 (C-6′), 66.6 (C-2′), 28.6 (C-3′), 23.4 (C-4′), and 53.2 (C-5′) suggested a proline moiety [46]. The remaining three hydrogen deficiencies and the COSY spin–spin coupling network from H-13 to H-9 and H-6 implied a eudesmane-type sesquiterpene lactone, eudesmanolide.

**Figure 4:**
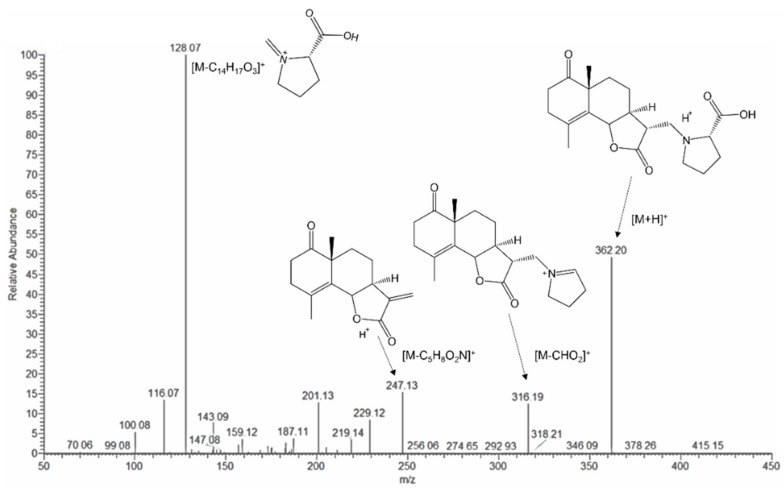
ESI-MS/MS spectrum (in-source CID fragmentation) of **1**.

The HMBC correlations (Figure 3) from H-2′ (δ_H_ 3.30, dd, *J* = 8.7, 4.6 Hz) to C-13, C-3′, and C-5′; from H-5′ to C-13 and C-2′; and from H-13 to C-12 and C-11 indicated the attachment of proline moiety at C-13 with N. The lower-shifted chemical shift value of C-13 (δ_C_ 51.2) was also consistent with this result. Further analysis on HMBC correlations from H-14 to C-10, C-9, C-5, and C-1; from H-2 (δ_H_ 2.55, ddd, *J* = 13.6, 7.0, 6.2 Hz, β; δ_H_ 2.43, ddd, *J* = 13.6, 7.8, 6.6 Hz, α) to C-3 and C-1; and from H-3 (δ_H_ 2.35, br dt, *J* = 17.0, 6.2 Hz, α; δ_H_ 2.26, br dt, *J* = 17.0, 6.9 Hz, β) to C-1, C-2, C-4, and C-5 suggested the position of the carbonyl group at C-1. The ESI-MS/MS spectrum (Figure 4) exhibited fragment ion peaks at *m*/*z* (relative intensity, %) 316 (13) and *m*/*z* 247 (15), resulting from the loss of a carboxy and a proline group from the molecular ion, respectively. The base ion peak *m*/*z* 128 (100) also agreed with the proline moiety. Acid hydrolysis of **1** with 1% HCl liberated L-proline by HPLC analysis with an optical rotation detector.

**Figure 5:**
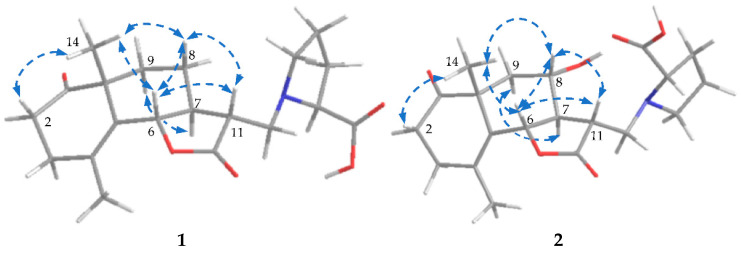
Selected NOESY correlations of compounds **1** and **2**.

To determine the absolute configuration of **1**, NOESY and CD spectra were measured. The NOESY correlations between H-6 and H-11, between H-14 and H-6, and between H-9α and H-7 revealed the relative configuration of **1**, as shown in Figure 5. In the CD spectra, the Cotton effects (Δε (nm): +4.62 (218), −4.70 (295), MeOH) were similar to the reported eudesmanolide, gracilin [47] (Δε (nm): +1.13 (223), −1.70 (294), MeOH), which indicated the absolute configuration should be 6S, 7S, 10R, 11R, 2′S. Accordingly, the structure of crossoseamine A (**1**) was determined as shown in Figure 1.

#### 2.1.2. Structure of Crossoseamine B (**2**)

Crossoseamine B (**2**) was isolated as colorless needles with a specific optical rotation (${\;[\alpha ]}_{D}^{27}-47,\;c\;0.46,\;\mathrm{MeOH}$). HR-ESI-MS data displayed a protonated molecule [M + H]^+^ at *m*/*z* 378.1912 (calcd for C_20_H_28_O_6_N: 378.1911), from which the molecular formula was determined as C_20_H_27_O_6_N suggesting an additional oxygen atom on **2** compared to **1**. The fragment ion peaks from ESI-MS/MS showing *m*/*z* 360 (34) [M + H-H_2_O]^+^, *m*/*z* 332 (19) [M-COOH]^+^ and 128 (100) [M-C_14_H_17_O_4_ (proline)]^+^ indicated a similarity to **1** (see Figure S23). The ^1^H and ^13^C NMR spectrum data were similar to compound **1**, except for the absence of one methylene carbon and the presence of an oxymethine carbon (δ_C_ 66.0) (Table 1). The HMBC correlations from H-7 (δ_H_ 1.88, m) to C-8 and C6 and from H-8 (δ_H_ 3.89, td, *J* = 10.8, 4.5 Hz) to C-6 and C-11 revealed that the position of oxymethine carbon (δ_C_ 66.0) was at C-8. The relative configuration was deduced as the same as **1** by NOESY analysis (Figure 5), where correlations between H-14 and H-6, between H-6 and H-11, between H-6 and H-8, and between H-14 and H-8 were observed. HPLC analysis of acid hydrolysate revealed the presence of L-proline. Ultimately, based on these spectroscopic data, HPLC analysis, and the similar Cotton effects with compound **1**, the structure of crossoseamine B (**2**) was established as 6S, 7R, 8S, 10R, 11R, 2′S-8-hydroxy analog of **1** as shown in Figure 1.

#### 2.1.3. Structure of 6′-O-Caffeoyl Scopolin (**3**)

**Figure 6:**
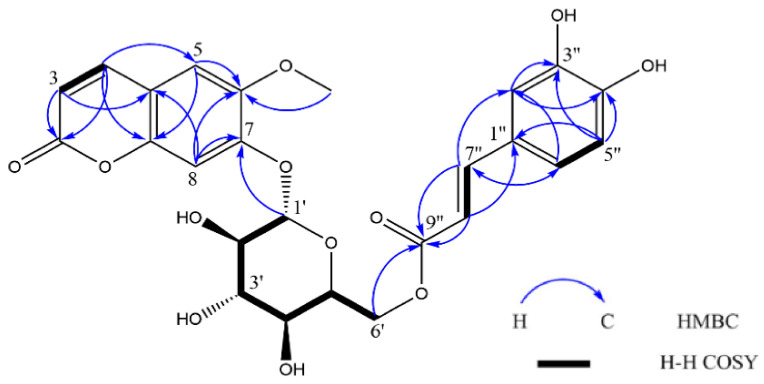
COSY and Key HMBC correlations of **3**.

Compound **3** was isolated as white amorphous powder with negative optical rotation (${\left[\alpha \right]}_{D}^{26}-76.5,\;c=0.2,\;\mathrm{MeOH}$). The molecular formula was determined as C_25_H_24_O_12_ from its HR-ESI-MS: *m*/*z* 539.1160 [M + Na]^+^ (calcd for C_25_H_24_O_12_Na: 539.1163). HPLC analysis with an optical rotation detector revealed that acid hydrolysis of **3** with 1M HCl at 80 °C released D-glucose. The results of ^1^H-NMR and ^13^C-NMR (Table 2) were similar to those published for 6′-O-sinapinoyl esculin, and indidenes F [48,49] (Table S1). The ^1^H-NMR spectrum showed several downfield doublets at δ_H_ 6.32 (1H, d, *J* = 9.5 Hz, H-3), δ_H_ 7.65 (1H, d, *J* = 9.5 Hz, H-4), δ_H_ 6.69 (1H, d, *J* = 15.9 Hz, H-8″), and δ_H_ 7.92 (1H, d, *J* = 15.9 Hz, H-7″), corresponding to cis- and trans-double bonds, respectively. Moreover, several characteristic low-field signals at δ_H_ 7.31 (1H, d, *J* = 8.3 Hz, H-5″), δ_H_ 7.570 (1H, d, *J* = 1.8 Hz, H-2″), and δ_H_ 7.27 (1H, dd, *J* = 8.3, 1.8 Hz, H-6″) were ascribed to a 1,3,4-trisubstituted benzene ring. In the ^13^C-NMR spectrum, two carbonyl carbons (δ_C_ 161.6 and 167.9), seven downfield quaternary carbons (δ_C_ 113.5, 127.1, 147.4, 147.8, 149.8, 150.8, and 151.5), one methoxy carbon (δ_C_ 56.5), five oxymethine carbons (δ_C_ 102.3, 78.7, 76.1, 74.9, and 71.6), and one oxymethylene carbon (δ_C_ 64.6), corresponding to D-glucose, were observed. HMBC correlations (Figure 6) from H-3 and H-4 to C-2; from H-3 to C-4a; from H-4 to C-8a and C-5; from H-5 (δ_H_ 7.01, 1H, s) to C-6 and C-8a; and from H-8 (δ_H_ 7.568, 1H, s) to C-4a, C-6, and C-7 indicated a coumarin moiety. Moreover, HMBC correlations from H-7″ and H-8″ to C-9″; from H-8″ to C-1″; and from H-7″ to C-2″ and C-6″, together with the characteristic 1,3,4-trisubstituted benzene coupling pattern of H-2″, H-5″, and H-6″, indicated the presence of a caffeoyl group. The coupling constant of the anomeric proton (δ_H_ 5.80, 1H, d, *J* = 7.3 Hz, H-1′) indicated the anomeric configuration of glucose should be β. Finally, the HMBC correlations from H-1′ to C-7 and from H-6′ (δ_H_ 4.91, dd, *J* = 11.9, 6.5 Hz; δ_H_ 5.11, dd, *J* = 11.9, 1.9 Hz) to C-9″ indicated the connection between coumarin, glucose, and the caffeoyl groups. Accordingly, the structure of **3** was defined as 6′-O-caffeoyl scopolin (Figure 1).

#### 2.1.4. Plausible Biosynthetic Pathway of **1** and **2**

**Figure 7:**
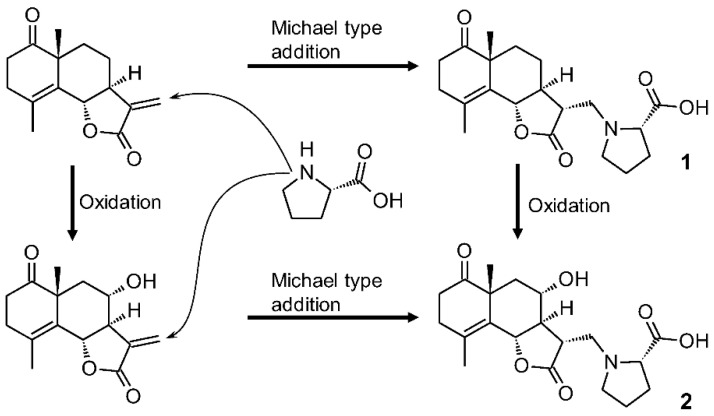
Plausible biosynthetic pathway of compounds **1** and **2**.

The plausible biosynthetic pathway of crossoseamines A and B (**1** and **2**) is the Michael-type reaction of the nitrogen of proline to the eudesmanolide moiety (Figure 7). Yoshikawa et al. (1993) have reported a chemical synthesis of similar proline conjugates by the reaction of α-methylene γ-lactone type eudesmanolide and proline with Et_3_N at 90 °C [50]. The demand for a relatively strong base and high temperature for chemical synthesis indicates the involvement of the biosynthetic enzyme to form **1** and **2** in *C. chinense*, not as artifacts through the extraction and purification processes. The 6,7-dihydroxy coumarin moiety of **3** is thought to be the lactone form of caffeic acid, i.e., esculetin, which undergoes further methylation, glycosylation, and caffeoylation to **3**. Given the other isolated compounds having caffeoyl function, *C. chinense* is considered a rich source of caffeic acid and its derivatives.

### 2.2. Cytotoxic Activities of the Isolated Compounds

All isolated compounds from the EtOAc fraction of *C. chinense* were evaluated for their cytotoxic activities against *L. major*, *P. falciparum*, *T. brucei* (gambiense and rhodesiense), and human lung cancer cell line A549 (Table 3).

The cytotoxicity against the human lung cancer cell line, A549, and the pathogen of cutaneous leishmaniasis, *L. major*, were evaluated by MTT method with positive control etoposide and miltefosine, respectively. The results are summarized in Table 3. Compounds **1** and **2** and some flavonoids (**6**–**8**) showed significant activities comparable to the positive controls.

Crossoseamine (**1**) and known compounds (**6**–**9**, **14**, **18**) had activities against both subspecies, *T. brucei gambiense* and *T. brucei rhodesiense* (Table 3), by ATP measurement using luciferase. Although the anti-*Trypanosoma* activity of compounds **6**–**9** and **18** was reported previously [32,33,34,35,36], this is the first time to show the potential of compound **14**.

The anti-*Plasmodium* activity was evaluated by the SYBR Green I method. The activity of compounds **1**, **2**, **11**, and **14** are reported for the first time, whereas that of compounds **6**–**9**, **12**, **13**, **15**, and **18** was previously reported [37,38,39,40]. The reported activities of the above compounds were consistent with our results, with some deviations (Table S2). The slight differences were ascribable to the differences in the assay methods and conditions and species/strains of the parasites used.

## 3. Experiments

### 3.1. General Experimental Procedure

Optical rotations were measured on a P-1030 spectropolarimeter (JASCO, Tokyo, Japan). IR and UV spectra were measured on FT-720 (HORIBA, Kyoto, Japan) and V-520 UV/Vis spectrophotometers (JASCO, Japan), respectively. ^1^H- and ^13^C-NMR spectra were measured on Avance Ⅲ HD spectrometer (Bruker, Billerica, MA, USA) at 500 and 125 MHz, respectively, with the residual solvent signal as references. Positive- and negative-ion HR-ESI-MSs were recorded on an LTQ Orbitrap XL spectrometer (Thermo Fisher Scientific, Waltham, MA, USA), and MS/MS fragments of precursor ions were detected by the CID mode with a collision energy of 35 eV.

Silica gel column chromatography (CC) and reversed-phase octadecyl silanized silica gel (ODS) CC were performed on silica gel 60 (Merck, Darmstadt, Germany) and Cosmosil 75C18-OPN (Nacalai Tesque, Kyoto, Japan). HPLC was performed on an Inertsil ODS-3 column (GL Science, Tokyo, Japan; Φ = 10 mm, L = 25 cm) or a Cosmosil πNAP column (Nacalai Tesque, Kyoto, Japan; Φ = 10 mm, L = 25 cm), and the eluate was monitored with a refractive index detector. TLC was performed on precoated silica gel 60 F_254_ plates (E. Merck; 0.25 mm in thickness). Sugars and proline were analyzed by HPLC on an amino and HILIC columns using a chiral detector (JASCO OR-2090 plus) (Asahipak NH_2_P-50 4E (Shodex, Tokyo, CH_3_CN-H_2_O (3:1), 1.0 mL/min); Cosmosil HILIC (Nacalai Tesque, Kyoto, Japan, CH_3_CN-H_2_O (4:1), 0.7 mL/min)).

### 3.2. Plant Material

The aerial parts of *C. chinense* were collected in July 2008 in Okinawa, Japan, and a voucher specimen was deposited in the Herbarium of the Department of Pharmacognosy, Graduate School of Biomedical Sciences, Hiroshima (deposition number: 08-CC-Okinawa-0708).

### 3.3. Extraction and Isolation

Air-dried aerial parts of *C. chinense* (3.5 kg) were extracted with MeOH (3 × 10 L) at room temperature. The methanol extract was concentrated to 1.5 L and then partitioned with an equal volume of n-hexane to obtain an n-hexane-soluble layer (27.7 g). The remaining layer was evaporated and resuspended in 1.5 L of water and then extracted with 1.5 L of EtOAc and 1.5 L of 1-BuOH successively to obtain EtOAC (74.3 g), 1-BuOH (30.5 g), and H_2_O (171.2 g) soluble fractions.

The EtOAC fraction (57.4 g of 74.3 g) was subjected to normal-phase open column CC (silica gel, Φ = 6, L = 25 cm) with increasing amounts of MeOH in CHCl_3_[(CHCl_3_, 2.0 L), CHCl_3_-MeOH (20:1, 2.0 L), CHCl_3_-MeOH (15:1, 2.0 L), CHCl_3_-MeOH (10:1, 2.0 L), CHCl_3_-MeOH (7:1, 2.0 L), CHCl_3_-MeOH (5:1, 2.0 L), CHCl_3_-MeOH (3:1, 2.0 L), CHCl_3_-MeOH (2:1, 2,0 L), and (MeOH, 2.0 L)] to obtain nine fractions [Fr. 1 (0.23 g), Fr. 2 (4.7 g), Fr. 3 (24.3 g), Fr. 4 (4.8 g), Fr. 5 (7.0 g), Fr. 6 (5.0 g), Fr. 7 (1.6 g), Fr. 8 (1.7 g), and Fr. 9 (4.1 g)] named CC-E 1 to CC-E 9. The fraction CC-E 3 (24.3 g) was separated by reversed-phase open column CC (ODS) with step gradient elution [(MeOH-H_2_O 2:3, 0.5 L), (MeOH-H_2_O 1:1, 0.5 L), (MeOH-H_2_O 3:2, 0.5 L), (MeOH-H_2_O 7:3, 0.5 L), (MeOH-H_2_O 4:1, 0.5 L), (MeOH-H_2_O 9:1, 0.5 L), (MeOH, 0.5 L), and (Acetone, 0.5 L)] to obtain eight subfractions [CC-E 3-1 (7.48 g), CC-E 3-2 (5.25 g), CC-E 3-3 (1.93 g), CC-E 3-4 (2.14 g), CC-E 3-5 (1.92g), CC-E 3-6, (1.28 g), CC-E 3-7, (1.05 g), and CC-E 3-8, (0.19 g)]. Of the 5.25 g of Fr. CC-E 3-2, 1.19 g was purified by ODS HPLC [Acetone-H_2_O (3:7, *v*/*v*)] to obtain scopoletin (**4**, 186.6 mg). Fr. CC-E 3-3 (1.93 g) was separated by ODS HPLC [Acetone-H_2_O (4.5:5.5, *v*/*v*)+ 0.1% TFA] to acquire chrysosplenol D (**6**, 107.4 mg). Fr. CC-E 4 (4.81 g) was fractionated by reversed-phase silica gel CC (ODS) with solvent [(MeOH-H_2_O 3:7, 0.5 L), (MeOH-H_2_O 2:3, 0.5 L), (MeOH-H_2_O 1:1, 0.5 L), (MeOH-H_2_O 3:2, 0.5 L), (MeOH-H_2_O 7:3, 0.5 L), (MeOH-H_2_O 4:1, 0.5 L), (MeOH-H_2_O 9:1, 0.5 L), (MeOH, 0.5 L), and (Acetone, 0.5 L)] to yield nine subfractions [CC-E 4-1 (0.78 g), CC-E 4-2 (0.38 g), CC-E 4-3 (0.61 g), CC-E 4-4 (0.51 g), CC-E 4-5 (0.36 g), and CC-E 4-6~ 4-9 (1.55 g)]. Subfraction CC-E 4-1 (0.78 g) was purified with πNAP HPLC [Acetone-H_2_O (3.5:6.5, *v*/*v*)] to obtain caffeic acid (**17**, 10.1 mg) and 3,4-dihydroxyl benzoic acid (**18**, 19.4 mg). CC-E 4-3 (0.61 g) [Acetone-H_2_O (2:3, *v*/*v*)] was separated by ODS HPLC to obtain 3-methylquercetin (**7**, 50.3 mg) and luteolin (**8**, 31.5 mg). Fr. CC-E 4-4 (0.51 g) was purified by ODS HPLC [Acetone-H_2_O (1:1, *v*/*v*)] to obtain 4,5-di-O-caffeoylquinic acid butyl ester (**11**, 32.4 mg), tianshic acid (**20**, 73.1 mg), and tianshic acid methyl ester (**21**, 8.6 mg). Fr. CC-E 5 (7.0 g) was fractionated by reversed-phase silica gel CC [(MeOH-H_2_O 3:7, 0.5 L), (MeOH-H_2_O 2:3, 0.5 L), (MeOH-H_2_O 1:1, 0.5 L), (MeOH-H_2_O 3:2, 0.5 L), (MeOH-H_2_O 7:3, 0.5 L), (MeOH-H_2_O 4:1, 0.5 L), (MeOH-H_2_O 9:1, 0.5 L), (MeOH, 0.5 L), and (Acetone, 0.5 L)] to afford nine subfractions [CC-E 5-1 (1.27 g), CC-E 5-2 (2.29 g), CC-E 5-3 (0.7 g), CC-E 5-4 (0.48 g), and CC-E 5-5~5-9 (1.8 g)]. Of the 2.29 g of Fr. CC-E 5-2, 1.29 g was purified by ODS HPLC [Acetone-H_2_O (3.5:6.5, *v*/*v*)] to acquire 6′-O-caffeoyl scopolin (**3**, 3.8 mg), 4,5-di-O-caffeoylquinic acid methyl ester (**12**, 41.3 mg), and 3,5-di-O-caffeoylquinic acid methyl ester (**13**, 46.7 mg). CC-E 5-4 (0.48 g) was separated by πNAP HPLC [Acetone-H_2_O (3.8:6.2, *v*/*v*)] to afford tetrecentronside B (**19**, 14.5 mg). Fraction CC-E 6 (5.03 g) was subjected to reversed-phase silica gel CC [(MeOH-H_2_O 1:4, 0.5 L), (MeOH-H_2_O 3:7, 0.5 L), (MeOH-H_2_O 2:3, 0.5 L), (MeOH-H_2_O 1:1, 0.5 L), (MeOH-H_2_O 3:2, 0.5 L), (MeOH-H_2_O 7:3, 0.5 L), (MeOH-H_2_O 4:1, 0.5 L), (MeOH-H_2_O 9:1, 0.5 L), (MeOH, 0.5 L), and (Acetone, 0.5 L)] to obtain ten subfractions [CC-E 6-1 (0.62 g), CC-E 6-2 (1.36 g), CC-E 6-3 (1.24 g), CC-E 6-4 (0.64 g), CC-E 6-5 (0.37 g), and CC-E 6-6~6-10 (1.02 g)]. Fr. CC-E 6-1 (0.62 g) was purified with πNAP HPLC [Acetone-H_2_O (1:4, *v*/*v*)] to obtain scopolin (**5**, 10.9 mg). CC-E 6-2 (1.36 g) was purified by πNAP HPLC [Acetone-H_2_O (1:4, *v*/*v*)] to obtain chlorogenic acid methyl ester (**15**, 33.6 mg) and 2,6-dimethoxyl-4-hydroxymethyl-phenol-1-O-(6-O-caffeoyl)-β-D-glucopyranoside (**16**, 15.1 mg). CC-E 6-3 (1.24 g) was purified with ODS HPLC [Acetone-H_2_O (2:3, *v*/*v*)] to obtain cosmoslin (**9**, 5.1 mg). CC-E 6-5 (0.37 g) was purified with πNAP HPLC [Acetone-H_2_O (2:3, *v*/*v*)] to afford 3,4,5-tri-O-caffeoylqunic acid methyl ester (**14**, 26.6 mg). Fraction CC-E 7 (1.63 g) was combined with CC-E 8 (1.68 g) and then separated by ODS CC [(MeOH-H_2_O 1:9, 0.5 L), (MeOH-H_2_O 1:4, 0.5 L), (MeOH-H_2_O 3:7, 0.5 L), (MeOH-H_2_O 2:3, 0.5 L), (MeOH-H_2_O 1:1, 0.5 L), (MeOH-H_2_O 3:2, 0.5 L), (MeOH-H_2_O 7:3, 0.5 L), (MeOH-H_2_O 4:1, 0.5 L), (MeOH-H_2_O 9:1, 0.5 L), (MeOH, 0.5 L), and (Acetone, 0.5 L)] to obtain eleven subfractions [CC-E 7-1 (0.15 g), CC-E 7-2 (0.12 g), CC-E 7-3 (0.26 g), CC-E 7-4 (0.98 g), and CC-E 7-5~7-11 (1.45 g)]. Fraction CC-E 7-3 (0.26 g) was purified with πNAP HPLC [Acetone-H_2_O (1.5:8.5, *v*/*v*)] to obtain crossoseamine B (**2**, 5.3 mg). CC-E 7-4 (0.98 g) was purified with πNAP HPLC [Acetone-H_2_O (1:1, *v*/*v*)] to obtain quercetin-3-O-(6″-O-α-rhamnosyl)-β-glucoside (**10**, 23.1 mg). Fraction CC-E 9 (4.13 g) was separated by reversed-phase silica gel CC [(MeOH-H_2_O 1:9, 0.5 L), (MeOH-H_2_O 1:4, 0.5 L), (MeOH-H_2_O 3:7, 0.5 L), (MeOH-H_2_O 2:3, 0.5 L), (MeOH-H_2_O 1:1, 0.5 L), (MeOH-H_2_O 3:2, 0.5 L), (MeOH-H_2_O 7:3, 0.5 L), (MeOH-H_2_O 4:1, 0.5 L), (MeOH-H_2_O 9:1, 0.5 L), (MeOH, 0.5 L), and (Acetone, 0.5 L)] to obtain eleven subfractions [CC-E 9-1 (1.49 g), CC-E 9-2 (0.16 g), CC-E 9-3 (0.63 g), and CC-E 9-4~9-11 (1.74 g)]. Fr. CC-E 9-3 (0.63 g) was purified with ODS HPLC [Acetone-H_2_O (1.8:8.2, *v*/*v*)] to obtain crossoseamine A (**1**, 3.1 mg).

The known compounds were identified by comparison of their physicochemical data, ${\;[\alpha ]}_{D}^{}$, IR, MS, ^1^H, and ^13^C NMR) with the reported data.

Crossoseamine A (**1**): a colorless amorphous powder, ${\;[\alpha ]}_{D}^{27}-97\;(c=0.2,\;\mathrm{MeOH}$); IR (film) ν_max_: 3411, 2943, 1762, 1710, 1633, 1450, 1374, 1319, 1225, 1038. UV λ_max_ (MeOH) nm (log ε): 205 (4.12); CD (MeOH) Δε (nm): +4.62 (218), −4.70 (295); ^1^H NMR (500 MHz, DMSO-d_6_), Table 1; ^13^C NMR (125 MHz, DMSO-d_6_), Table 1; positive-ion HR-ESI-MS: *m*/*z* 362.1962 [M + H]^+^ (calcd for C_20_H_28_O_5_N: 362.1961); ESI MS/MS: *m*/*z* 362 [M + H]^+^ (48), 316 [M–COOH]^+^ (12), 247 [M–C_5_H_8_O_2_N (proline)]^+^ (15), 128 [M–C_14_H_17_O_3_ (eudesmanolide part)]^+^ (100).

Crossoseamine B (**2**): colorless needles, ${\;[\alpha ]}_{D}^{27}-47\;(c=0.46,\;\mathrm{MeOH}$); mp. 210–214 °C; IR (film) ν_max_: 3248, 2980, 1758, 1711, 1625, 1450, 1379, 1310, 1033. UV λ_max_ (MeOH) nm (log ε): 204 (4.13); CD (MeOH) Δε (nm): +1.84 (233), −4.61 (294); ^1^H NMR (500 MHz, DMSO-d_6_), Table 1; ^13^C NMR (125 MHz, DMSO-d_6_), Table 1; positive-ion HR-ESI-MS: *m*/*z* 378.1912 [M + H]^+^ (calcd for C_20_H_28_O_6_N: 378.1911); ESI-MS/MS: *m*/*z* 378 [M + H]^+^ (66), 360 [M + H-H_2_O]^+^ (34), *m*/*z* 332 [M-COOH]^+^ (19) and 128 [M-C_14_H_17_O_4_ (eudesmanolide part)]^+^ (100).

6′-O-caffeoyl scopolin (**3**): a colorless amorphous powder, ${\;[\alpha ]}_{D}^{27}-76.5\;(c=0.2,\;\mathrm{MeOH})$; IR (film) ν_max_: 3393, 2952, 2849, 1689, 1639, 1511, 1389, 1278, 1164, 1019. UV λ_max_ (MeOH) nm (log ε) 225sh (3.92), 250sh (3.62), 292 (3.66), 331 (3.75); ^1^H NMR (500 MHz, pyridine-d_5_), Table 2; ^13^C NMR (125 MHz, pyridine-d_5_), Table 2; positive-ion HR-ESI-MS: *m*/*z* 539.1160 [M + Na]^+^ (calcd for C_25_H_24_O_12_Na: 539.1163). ESI-MS/MS: *m*/*z* 521 [M + Na–H_2_O]^+^ (4), *m*/*z* 377 [M + Na–C_9_H_6_O_3_ (caffeoyl)]^+^ (5), *m*/*z* 347 [M + Na-C_10_H_8_O_4_ (scopoletin)]^+^ (100) and 215 [M + Na–C_15_H_16_O_8_ (6-O-caffeoylglucose]^+^ (29).

### 3.4. Acid Hydrolysis of Compounds **1** and **2**

Compounds **1** and **2** (0.2 mg each) were treated with 1% aqueous hydrochloric acid (HCl) (0.5 mL) at room temperature for 12 h. The reaction mixture was extracted with EtOAc to obtain the EtOAc and aqueous layers. The latter was subjected to HPLC analysis with an optical rotation detector (OR-2090 plus; JASCO) on a HILIC column (Cosmosil HILIC, 10 × 250 mm, CH_3_CN-H_2_O (4:1, *v*/*v*), flow rate: 0.7 mL/min). The peaks from **1** and **2** were identical with an authentic standard, L-proline (t_R_: 30.0 min, negative optical rotation) [51].

### 3.5. Sugar Analysis of Compound **3**

Compound **3** (0.5 mg) was hydrolyzed with 1M HCl (0.2 mL) at 80 °C for 2 h. After cooling, EtOAc was used to extract the reaction mixture, and the aqueous layer was subjected to HPLC analysis with an optical rotation detector (OR-2090 plus; JASCO) on an amino column [Asahipak NH_2_P-50 4E, 4.6 × 250 mm, CH_3_CN-H_2_O (3:1, *v*/*v*), flow rate: 1 mL/min] to identify the D-glucose from **3**, which was determined by comparison of its retention time and optical rotation sign with authentic sample (t_R_: 7.3 min, positive optical rotation).

### 3.6. Growth Inhibition Activity

The evaluation of cytotoxic activities against A549, *L. major*, *P. falciparum*, and *T. brucei* (*gambiense* and *rhodesiense*) was conducted following previous reports.

In brief, the human lung cancer cell line A549 was maintained in 10% FCS-supplemented DMEM. Various concentrations of samples in dimethylsulfoxide (DMSO) and A549 (5 × 10^3^ cells/well) were cultured in a CO_2_ incubator for 72 h. The medium was replaced with 100 µL of MTT solution and incubated for 1.5 h in the same condition. The viability was calculated from the absorbance of formed MTT formazan at 550 nm using a microplate reader [52]. Etoposide was used as a positive control.

The leishmanicidal activities of the isolated compounds were also evaluated using an MTT assay. In a 96-well plate, various concentrations of sample solutions in dimethylsulfoxide (DMSO) and *L. major* (2 × 10^5^ parasite/well) in 100 µL of M199 medium were incubated for 72 h at 25 °C. Then, 100 µL of MTT solution was replaced and incubated overnight. The absorbance of the formazan solution in DMSO was recorded using a microplate reader at 550 nm [11]. Miltefosine was used as a positive control.

The trypanocidal activities of the isolated compounds were performed in 96-well plates with slight modifications [53]. In brief, each well contains 100 µL of parasite culture (1 × 10^4^ parasites/well) with serial dilutions of compounds. After incubation for 72 h at 37 °C under 5% CO_2_, 25 µL of CellTiter-GloTM Luminescent Cell Viability Assay reagent (Promega Japan, Tokyo, Japan) was added to evaluate intracellular ATP concentration according to the instruction. Nifurtimox was used as a positive control.

Anti-*Plasmodium* activity of the isolated compounds was evaluated according to the previous report [54]. In brief, 100 µL of *P. falciparum* 3D7 parasite culture [55] was plated in a 96-well plate with various concentrations of the compounds. After incubation for 72 h at 37 °C in a humidified chamber under a gas mixture of 90% N_2_, 5% O_2,_ and 5% CO_2_, the parasitemia was determined by SYBR Green I assay (Lonza Ltd., Basel, Switzerland) with a microplate reader at 485 and 530 nm. Artemisinin dissolved in DMSO was used as a positive control, and DMSO was used as a negative control. Human erythrocytes and plasma were obtained from the Nagasaki Red Cross Blood Center, and their usage was approved by the ethical committee of the Institute of Tropical Medicine, Nagasaki University.

The 50% inhibitory concentration (IC_50_) values were obtained for each compound by linear regression method.

## 4. Conclusions

The ethyl acetate extract of *C. chinense* was intensively fractionated to obtain previously undescribed sesquiterpene-amino acid conjugate crossoseamines A and B (**1** and **2**) and a coumarin glucoside, 6′-*O*-caffeoyl scopolin (**3**) together with 18 known compounds (**4**–**21**). The plausible biosynthetic pathway of crossoseamines A and B (**1** and **2**) is the Michael-type reaction of the nitrogen of proline to the eudesmanolide moiety (Figure 7).

Crossoseamine A (**1**) showed potent activity against A549 and *L. major*, while the introduction of the hydroxy function on position 8 (crossoseamine B (**2**)) decreased the activity. The polarity and/or functionality of eudesmane moiety may be necessary for the cytotoxicity against these targets. Among the evaluated compounds, chrysosplenol D (**6**) showed substantial toxicity for all tested organisms, probably by general toxicity, and has already been reported previously [21,25,56]. While some flavonoids (**7**–**9**) and caffeic acid derivatives (**14**) are relatively specific for both *Trypanosoma* and *Plasmodium*, compounds (**11**–**13**, **15**, **18**) showed specificity for *Plasmodium*. As a result, these compounds have the potential to treat human cancer, leishmaniasis, malaria, and trypanosomiasis. However, further chemical modification and mechanism analyses are needed in the future.

## Figures and Tables

**Table 1:** ^13^C-NMR and ^1^H-NMR spectroscopic data for compounds **1** and **2**.

	1		2	
Position	^13^C	^1^H	^13^C	^1^H
1	212.9		212.2	
2	35.5	2.43 (*ddd*, 13.6, 7.8, 6.6) *α*	35.2	2.40 (*m*) *α*
		2.55 (*ddd*, 13.6, 7.0, 6.2) *β*		2.58 (*m*) *β*
3	32.3	2.35 (br *dt*, 17.0, 6.2) *α*	32.4	2.37 *(m) α*
		2.26 (br *dt*, 17.0, 6.9) *β*		2.27 (*m*) *β*
4	125.2		126.1	
5	130.0		128.6	
6	80.8	4.80 (*dquint*-like, 11.2, 1.3)	77.9	4.87 (*dquint*-like, 11.4, 1.1)
7	48.5	2.02 (*m*)	57.6	1.88 (*m*)
8	23.1	1.95 (*m*) *α*	66.0	3.89 (*td*, 10.8, 4.5)
		1.63 (*m*) *β*		
9	34.6	1.44 (*td*, 14.0, 5.1) *α*	43.1	1.25 (br *t*, 11.8) *α*
		1.63 (*m*) *β*		1.87 (*m*) *β*
10	48.4		47.4	
11	44.2	2.73 (*dt-*like, 12.1, 5.4)	44.3	3.01 (*m*)
12	177.0		175.8	
13	51.2	2.86 (*dd*, 13.2, 5.1)	52.8	2.92 (*m*)
		3.08 (*dd*, 13.2, 5.7)		3.15 (*m*)
14	23.0	1.25 (3H, *s*)	24.1	1.24 (3H, *s*)
15	19.4	1.86 (3H, br *s*)	19.3	1.87 (3H, br *s*)
2′	66.6	3.30 (*dd*, 8.7, 4.6)	66.3	3.45 (*dd*, 9.0, 3.4)
3′	28.6	1.81 (*m*)	28.9	1.90 (*m*)
		2.03 (*m*)		2.07 (*dq*-like, 12.8, 8.8)
4′	23.4	1.68–1.79 (2H, *m*)	23.1	1.74 (*m*)
				1.80 (*m*)
5′	53.2	2.58 (*dd*-like, 9.0, 7.8)	52.8	2.84 (*dt*, 9.5, 7.5)
		2.99 (*ddd*, 9.0, 7.5, 3.9)		3.00 (*m*)
6′	173.9		173.6	

500 MHz and 125 MHz (DMSO-d_6_). Chemical shifts (δ) in ppm. *m*: multiplet or overlapped signals.

**Table 2:** ^13^C-NMR and ^1^H-NMR spectroscopic data for **3**.

3					
Position	^13^C	^1^H	Position	^13^C	^1^H
2	161.6		4′	71.6	4.24 (br *t*, 8.8)
3	114.5	6.32 (*d*, 9.5)	5′	76.1	4.37 (*ddd*, 9.5, 6.5, 1.9)
4	144.0	7.65 (*d*, 9.5)	6′	64.6	4.91 (*dd*, 11.9, 6.5)
4a	113.5				5.11 (*dd*, 11.9, 1.9)
5	110.2	7.01 (*s*)	1″	127.1	
6	147.4		2″	116.5	7.570 (*d*, 1.8)
7	151.5		3″	147.8	
8	104.6	7.568 (*s*)	4″	149.8	
8a	150.8		5″	117.3	7.31 (*d*, 8.3)
6-OCH_3_	56.5	3.69 (3H, *s*)	6″	122.5	7.27 (*dd*, 8.3, 1.8)
1′	102.3	5.80 (*d*, 7.3)	7″	146.6	7.92 (*d*, 15.9)
2′	74.9	4.42 (*dd*-like, 8.8, 7.3)	8″	114.9	6.69 (*d*, 15.9)
3′	78.7	4.44 (br *t*, 8.8)	9″	167.9	

500 MHz and 125 MHz (pyridine-*d*_5_). Chemical shifts (*δ*) in ppm.

**Table 3:** IC_50_ of the isolated compounds against A549 and parasites (Mean ± SE, µg/mL, *n* = 3).

Compounds	A549	*L. major*	*T. b.* *gambiense*	*T. b.* *rhodesiense*	*P. falciparum*
**1**	3.3 ± 0.3	6.9 ± 0.6	26.3 ± 8.5	27.8 ± 10.4	12.1 ± 1.1
**2**	12.3 ± 1.0	24.9 ± 2.2	39.5 ± 6.9	41.0 ± 8.9	15.6 ± 1.2
**6**	4.4 ± 1.5	5.4 ± 0.8	2.6 ± 0.1	1.7 ± 0.3	6.0 ± 1.1
**7**	17.7 ± 4.4	17.5 ± 1.2	4.0 ± 0.2	3.2 ± 0.3	16.5 ± 1.1
**8**	19.9 ± 5.5	26.9 ± 2.8	4.9 ± 0.3	3.2 ± 0.4	11.0 ±1.3
**9**	78.3 ± 15.6	58.9 ± 7.5	16.9 ± 0.7	8.6 ± 1.2	8.9 ± 1.0
**10**	>100	90.9 ± 5.5	>100	>100	37.0 ± 1.1
**11**	73.5 ± 13.0	>100	47.4 ± 2.7	34.8 ± 4.6	5.4 ± 1.1
**12**	115.0 ± 19.3	>100	97.1 ± 0.2	68.9 ± 14.2	4.6 ± 1.1
**13**	118.2 ± 19.7	>100	>100	80.5 ± 18.4	10.4 ± 1.2
**14**	42.6 ± 6.9	58.3 ± 6.6	11.0 ± 0.1	12.4 ± 0.8	3.6 ± 1.1
**15**	79.4 ± 8.7	>100	83.8 ± 13.3	53.6 ± 2.2	10.2 ± 1.1
**16**	>100	>100	>100	>100	35.5 ± 1.0
**17**	42.5 ± 0.1	>100	53.2 ± 7.5	42.3 ±2.9	25.0 ± 1.1
**18**	>100	>100	19.6 ± 2.7	24.8 ± 1.2	15.9 ± 1.2
**19**	>100	>100	>100	55.2 ± 0.8	30.6 ± 1.1
**20**	>100	>100	44.1 ± 3.0	32.8 ± 1.8	26.1 ± 1.1
**21**	>100	>100	58.7 ± 3.0	36.0 ± 0.9	27.1 ± 1.1
PC	17.5 ± 0.3	7.4 ± 0.7	1.3 ± 0.7	1.3 ± 0.5	0.0023 ± 0.0003

PC (Positive control): A549 (Etoposide); *L. major* (Miltefosine); *T. b. gambiense/rhodesiense* (Nifurtimox). *P. falciparum* (Artemisinin). Compounds **3**–**5** were not active at 100 μg/mL and omitted.

## Data Availability

Data is contained within the article or supplementary material.

## Supplementary Materials

The following are available online at https://www.mdpi.com/article/10.3390/molecules28124696/s1, Figures S1–S32: 1D, 2D NMR spectra, MS and CD data of compounds **1**–**3**. Table S1: Comparison of NMR data of 3 with related compounds **A** and **B**. Table S2: Reported IC50 values of the identified known compounds.

## Sample Availability

Samples of the compounds **1**–**3** are available from the authors.
