# Supplementary material for: Two New Cytotoxic Sesquiterpene-Amino Acid Conjugates and a Coumarin-Glucoside from Crossostephium chinense
Source: Molecules. 2023 Jun 11;28(12):4696. doi: 10.3390/molecules28124696 (PMC10304467; doi:10.3390/molecules28124696)
Supplement: Supplementary file 1 [file molecules-28-04696-s001.zip › molecules-2438741-supplementary.pdf]

Supplementary Data

Two New Cytotoxic Sesquiterpene-Amino Acid Conjugates and a Coumarin-Glucoside from *Crossostephium chinense*

Zhichao Wang [1], Ben-Yeddy Abel Chitama [2], Keisuke Suganuma [3], Yoshi Yamano [1], Sachiko Sugimoto [1], Susumu Kawakami [4], Osamu Kaneko [2], Hideaki Otsuka [4] and Katsuyoshi Matsunami [1,*]

[1] Graduate School of Biomedical & Health Sciences, Hiroshima University, 1-2-3 Kasumi, Minami-ku, Hiroshima 734-8553, Japan; zhichao96425@gmail.com (Z.W.); yamano@hiroshima-u.ac.jp (Y.Y.); ssugimot@hiroshima-u.ac.jp (S.S.)

[2] Department of Protozoology, Institute of Tropical Medicine (NEKKEN), Nagasaki University, 1-12-4 Sakamoto, Nagasaki 852-8523, Japan; benchitama@gmail.com (B.-Y.A.C.); okaneko@nagasaki-u.ac.jp (O.K.)

[3] National Research Center for Protozoan Diseases, Obihiro University of Agriculture and Veterinary Medicine, Inada, Obihiro, Hokkaido 080-8555, Japan; k.suganuma@obihiro.ac.jp

[4] Graduate School of Pharmacy, Yasuda Women's University, Hiroshima 731-0153, Japan; kawakami@yasuda-u.ac.jp (S.K.); otsuka-h@yasuda-u.ac.jp (H.O.)

* Correspondence: matunami@hiroshima-u.ac.jp; Tel.: +81-82-257-5335

Contents:

Figure S1: ^{1}H NMR of compound **1** in 500 MHz, DMSO-d_6.
Figure S2: ^{1}H NMR (4.80 ppm) of compound **1** in 500 MHz, DMSO-d_6.
Figure S3: ^{13}C NMR of compound **1** in 125 MHz, DMSO-d_6.
Figure S4: ^{13}C DEPT NMR of compound **1** in 125 MHz, DMSO-d_6.
Figure S5: COSY of compound **1** in 500 MHz, DMSO-d_6.
Figure S6: HSQC of compound **1** in 125 & 500 MHz, DMSO-d_6.
Figure S7: HMBC (4Hz) of compound **1** in 125 & 500 MHz, DMSO-d_6.
Figure S8: HMBC (10Hz) of compound **1** in 125 & 500 MHz, DMSO-d_6.
Figure S9: PS-NOESY of compound **1** in 500 MHz, DMSO-d_6.
Figure S10: HR-ESI-MS of compound **1**.
Figure S11: ESI-MS/MS of compound **1**.
Figure S12: CD spectrum of compound **1**, MeOH.
Figure S13: ^{1}H NMR of compound **2** in 500 MHz, DMSO-d_6.
Figure S14: ^{1}H NMR (4.80 ppm) of compound **2** in 500 MHz, DMSO-d_6.
Figure S15: ^{13}C NMR of compound **2** in 125 MHz, DMSO-d_6.
Figure S16: ^{13}C DEPT NMR of compound **2** in 125 MHz, DMSO-d_6.
Figure S17: COSY of compound **2** in 500 MHz, DMSO-d_6.
Figure S18: HSQC of compound **2** in 125 & 500 MHz, DMSO-d_6.
Figure S19: HMBC (4Hz) of compound **2** in 125 & 500 MHz, DMSO-d_6.
Figure S20: HMBC (10Hz) of compound **2** in 125 & 500 MHz, DMSO-d_6.
Figure S21: PS-NOESY of compound **2** in 500 MHz, DMSO-d_6.
Figure S22: HR-ESI-MS of compound **2**.
Figure S23: ESI-MS/MS of compound **2**.
Figure S24: CD spectrum of compound 2, MeOH.
Figure S25: ^{1}H NMR of compound **3** in 500 MHz, Pyridine-d_5.
Figure S26: ^{13}C NMR of compound **3** in 125 MHz, Pyridine-d_5.
Figure S27: ^{13}C DEPT NMR of compound **3** in 125 MHz, Pyridine-d_5.
Figure S28: COSY of compound **3** in 500 MHz, Pyridine-d_5.
Figure S29: HSQC of compound **3** in 125 & 500 MHz, Pyridine-d_5.
Figure S30: HMBC (10Hz) of compound **3** in 125 & 500 MHz, Pyridine-d_5.
Figure S31: HR-ESI-MS of compound **3**.
Figure S32: ESI-MS/MS of compound **3**.
Table S1: Comparison of NMR data of **3** with related compounds **A** and **B**.
Table S2: Reported IC_{50} values of the identified known compounds.

Figure S1: ^{1}H NMR of compound **1** in 500 MHz, DMSO-d_6.

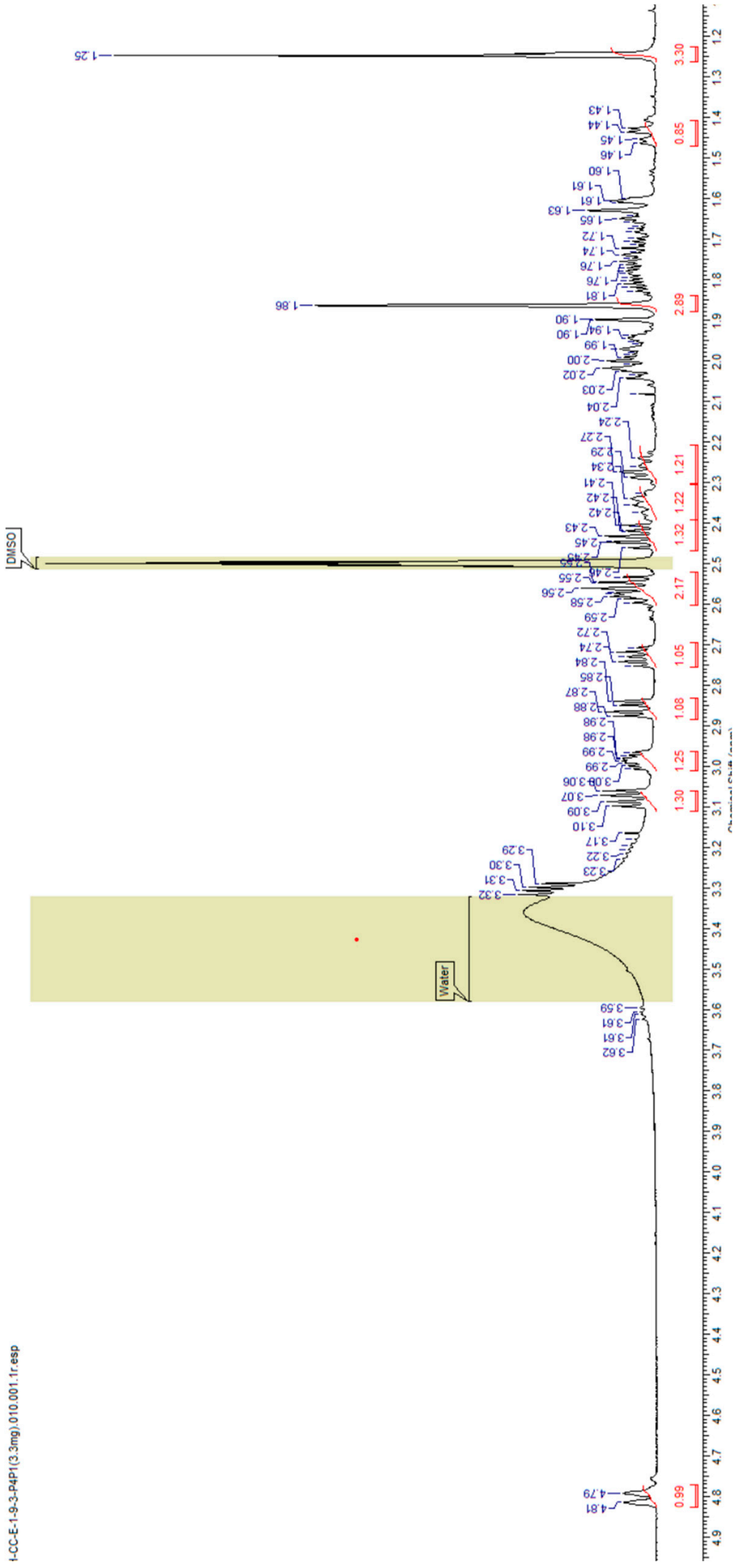

Current Data Parameters
NAME 20210925-Wang-CC-E-1-9-3-P4P1(3.3mg)
F2 - Acquisition Parameters

Date_	20210925
Time	13.02 h
INSTRUM	spect
PROBHD	Z113652_0220 (
PULPROG	zg30
TD	65536
SOLVENT	DMSO
NS	16
DS	2
SWH	7500.000 Hz
FIDRES	0.228882 Hz
AQ	4.3690667 sec
RG	110.57
DW	66.667 usec
DE	12.08 usec
TE	297.6 K
D1	1.00000000 sec
TD0	1
SFO1	500.1332508 MHz
NUC1	1H
P0	3.33 usec
P1	10.00 usec
PLW1	19.48699951 W

Figure S2: ^{1}H NMR (4.80 ppm) of compound **1** in 500 MHz, DMSO-d_6.

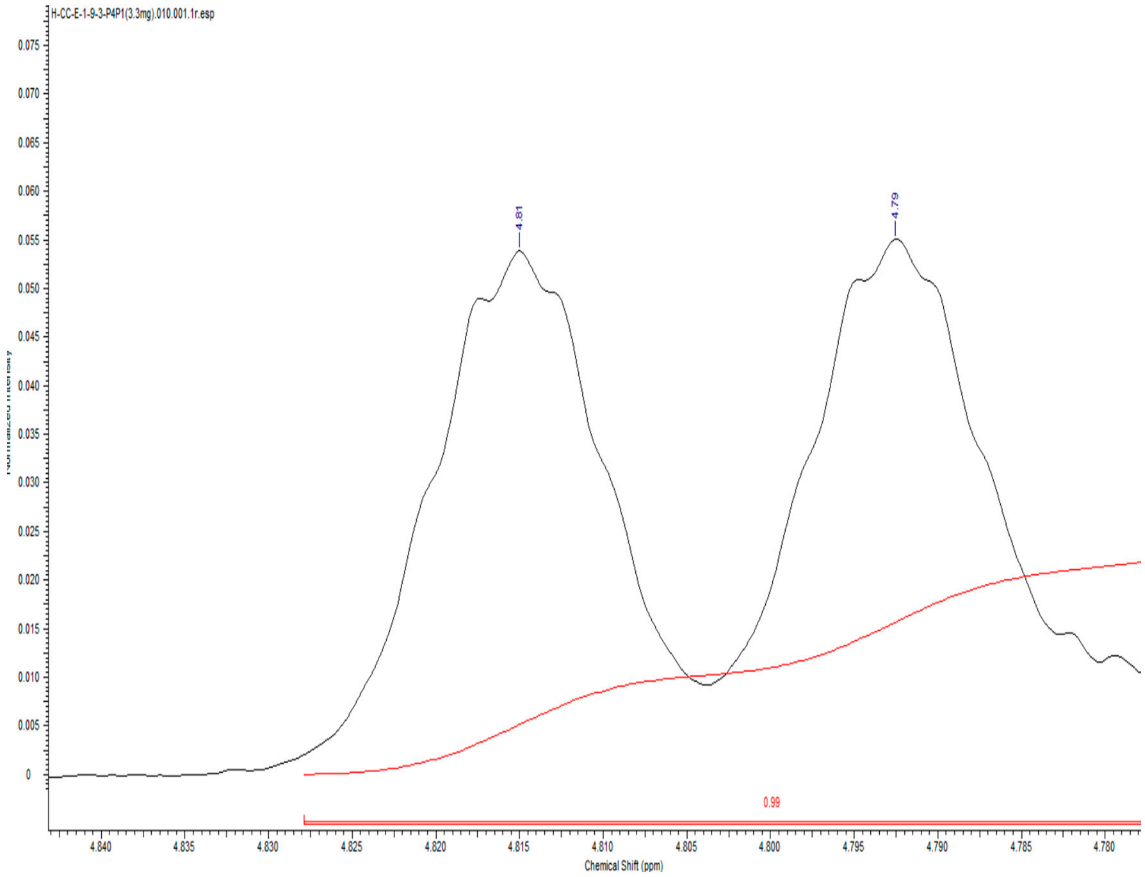

Figure S3: ^{13}C NMR of compound **1** in 125 MHz, DMSO-d_6.

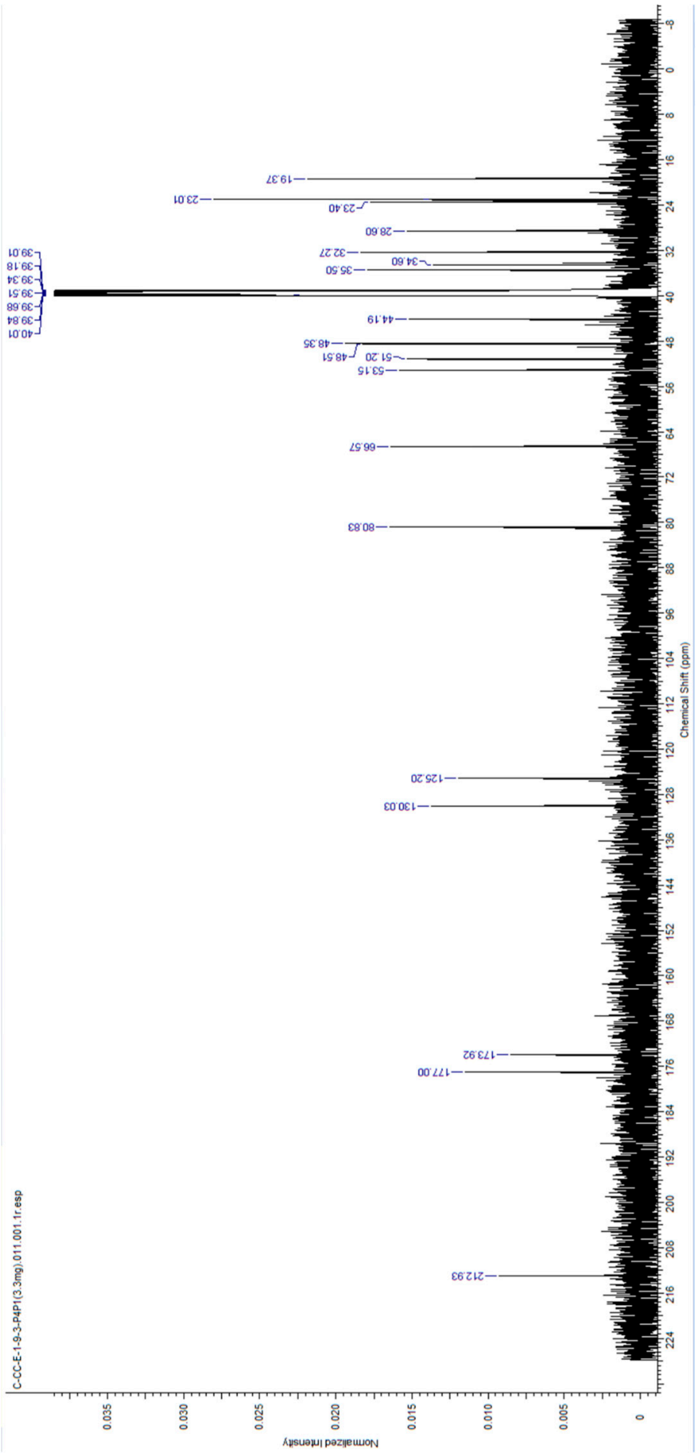

Current Data Parameters

NAME	20210925-Wang-CC-E-1-9-3-P4P1(3.3mg)
F2 - Acquisition Parameters	
Date_	20210925
Time	13.43 h
INSTRUM	spect
PROBHD	Z113652_0220 (
PULPROG	zgpg30
TD	65536
SOLVENT	DMSO
NS	747
DS	4
SWH	29761.904 Hz
FIDRES	0.908261 Hz
AQ	1.1010048 sec
RG	186.63
DW	16.800 usec
DE	6.50 usec
TE	298.6 K
D1	2.00000000 sec
D11	0.03000000 sec
TD0	1
SFO1	125.7716219 MHz
NUC1	13C
P0	3.33 usec
P1	10.00 usec
PLW1	85.47299957 W
SFO2	500.1320005 MHz
NUC2	1H
CPDPRG[2	waltz65
PCPD2	80.00 usec
PLW2	19.48699951 W
PLW12	0.30447999 W
PLW13	0.15315001 W

Figure S4: ^{13}C DEPT NMR of compound **1** in 125 MHz, DMSO-d_6.

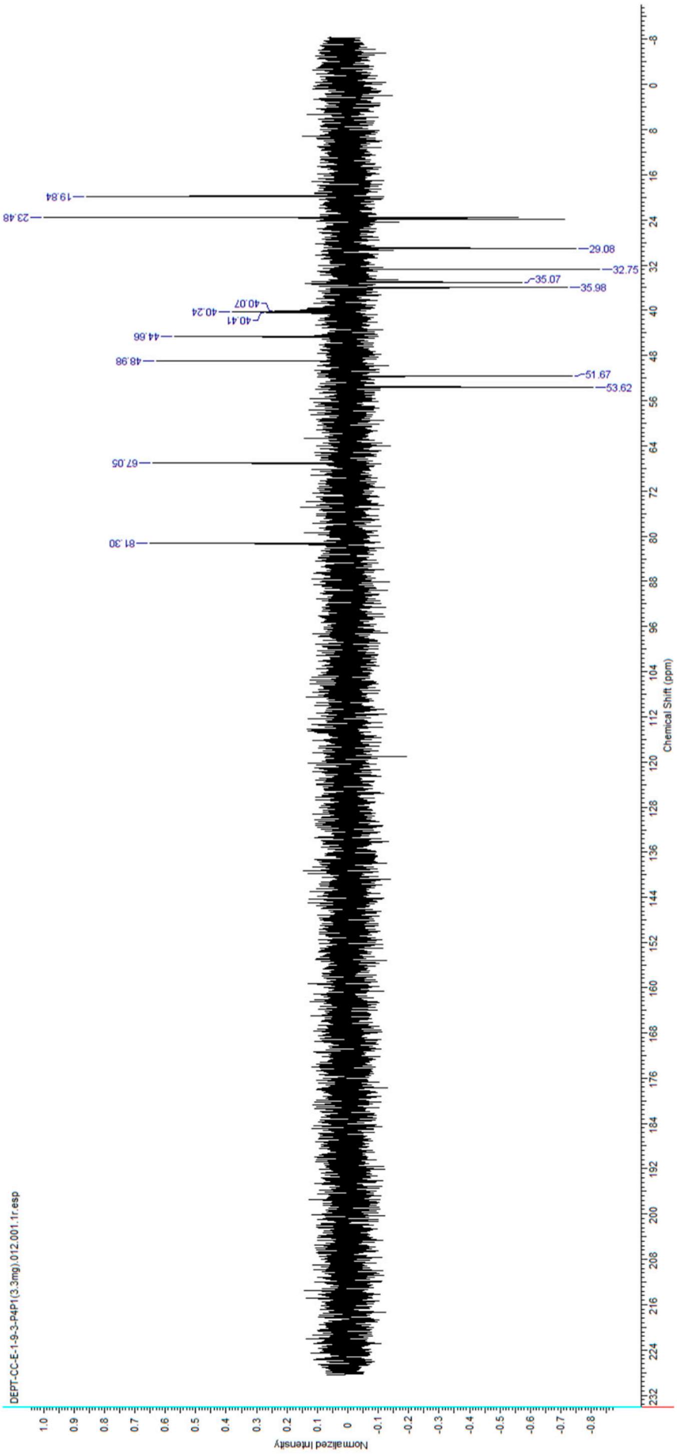

Current Data Parameters

NAME	20210925-Wang-CC-E-1-9-3-P4P1(3.3mg)
F2 - Acquisition Parameters	
Date_	20210925
Time	13.51 h
INSTRUM	spect
PROBHD	Z113652_0220 (
PULPROG	deptsp135
TD	65536
SOLVENT	DMSO
NS	112
DS	8
SWH	29761.904 Hz
FIDRES	0.908261 Hz
AQ	1.1010048 sec
RG	186.63
DW	16.800 usec
DE	6.50 usec
TE	298.2 K
CNST2	145.0000000
D1	2.00000000 sec
D2	0.00344828 sec
D12	0.00002000 sec
TD0	1
SFO1	125.7716219 MHz
NUC1	13C
P1	10.00 usec
P13	2000.00 usec
PLW0	0 W
PLW1	85.47299957 W
SPNAM[5]	Crp60comp.4
SPOAL5	0.500
SPOFFS5	0 Hz
SPW5	13.05900002 W
SFO2	500.1320005 MHz
NUC2	1H
CPDPRG[2	waltz65
P3	10.00 usec
P4	20.00 usec
PCPD2	80.00 usec
PLW2	19.48699951 W
PLW12	0.30447999 W

Figure S5: COSY of compound **1** in 500 MHz, DMSO-d_6.

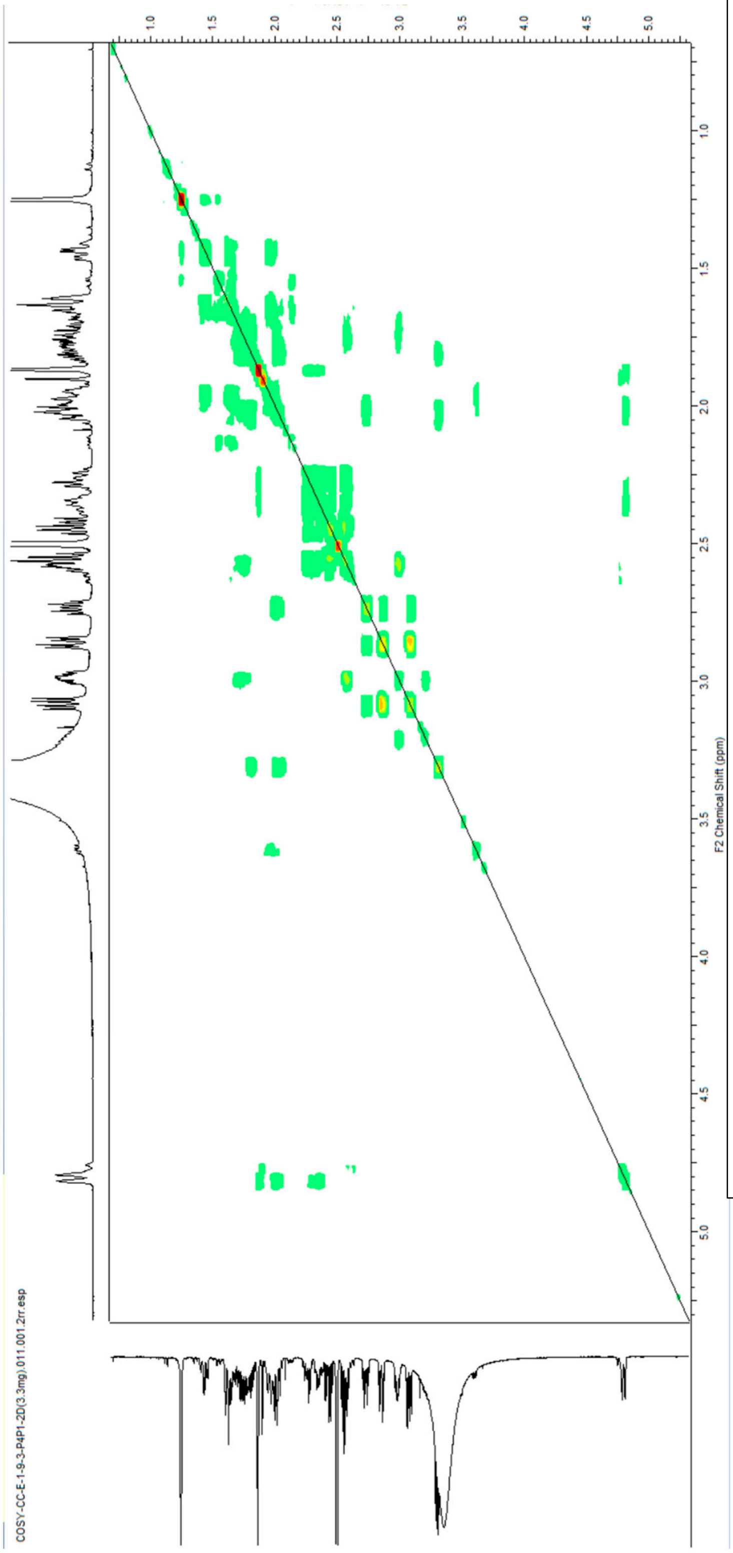

Current Data Parameters	
NAME	20210926-Wang-CC-E-1-9-3-P4P1(3.3mg)
F2 - Acquisition Parameters	
Date_	20210926
Time	9.52 h
INSTRUM	spect
PROBHD	Z113652_0220 (
PULPROG	cosygpppqf
TD	2048
SOLVENT	DMSO
NS	4
DS	16
SWH	2325.581 Hz
FIDRES	2.271075 Hz
AQ	0.4403200 sec
RG	61.94
DW	215.000 usec
DE	6.50 usec
TE	297.7 K
D0	0.00000300 sec
D1	1.69621301 sec
D11	0.03000000 sec
D12	0.00002000 sec
D13	0.00000400 sec
D16	0.00020000 sec
IN0	0.00043000 sec
TDav	1
SFO1	500.1315051 MHz
NUC1	1H
P0	10.00 usec
P1	10.00 usec
P17	2500.00 usec
PLW1	19.48699951 W
PLW10	2.16520000 W
GPNAM[1]	SMSQ10.100
GPZ1	10.00 %
P16	1000.00 usec

Figure S6: HSQC of compound **1** in 125 & 500 MHz, DMSO-d_6.

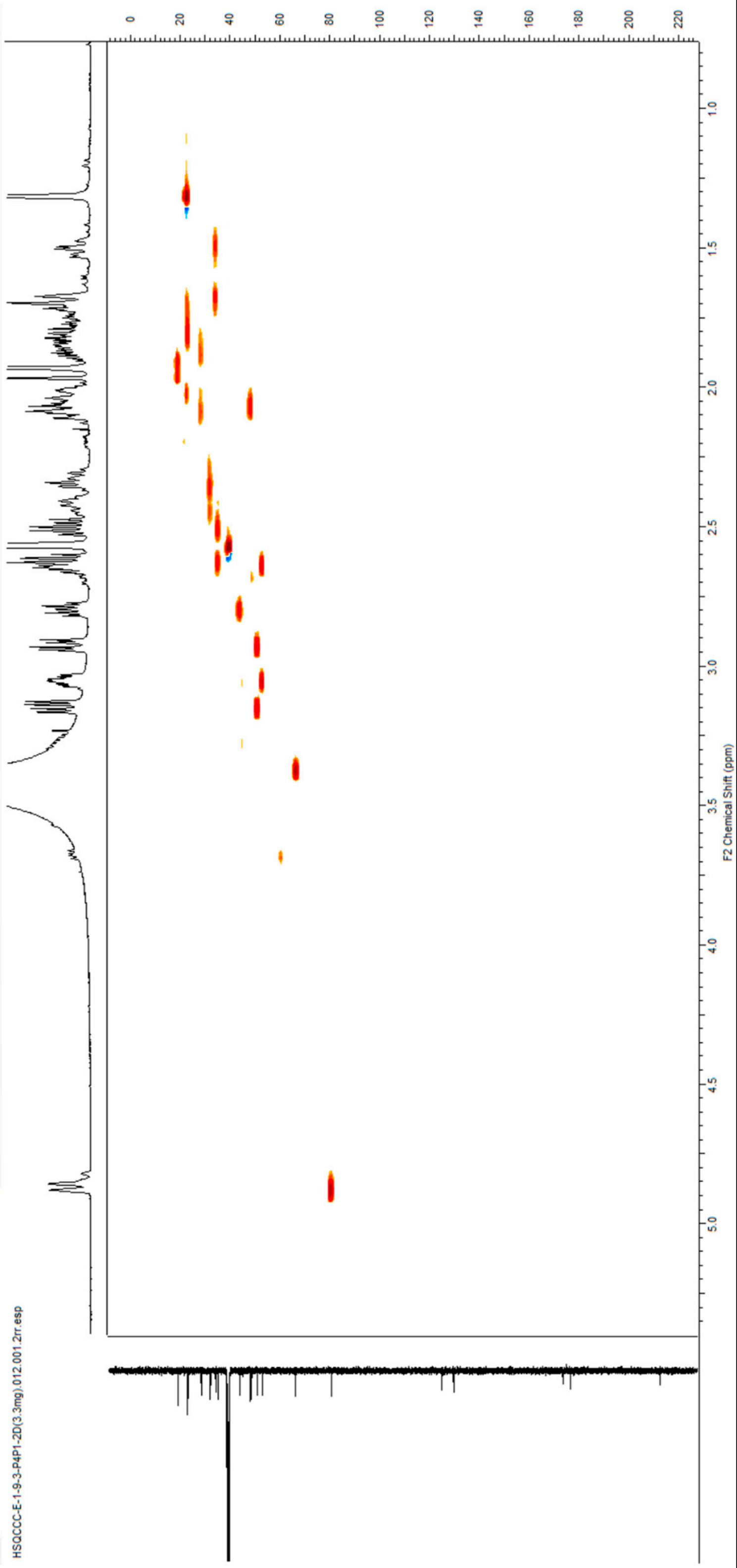

Current Data Parameters

NAME	20210926-Wang-CC-E-1-9-3-P4P1(3.3mg)

F2 - Acquisition Parameters

Date_	20210926
Time	10.12 h
INSTRUM	spect
PROBHD	Z113652_0220 (
PULPROG	hsqcetgpsi2
TD	512
SOLVENT	DMSO
NS	4
DS	16
SWH	2325.581 Hz
FIDRES	9.084302 Hz
AQ	0.1100800 sec
RG	186.63
DW	215.000 usec
DE	20.25 usec
TE	298.0 K
CNST2	145.0000000
D0	0.00000300 sec
D1	1.50000000 sec
D4	0.00172414 sec
D11	0.03000000 sec
D16	0.00020000 sec
D24	0.00086207 sec
IN0	0.00001680 sec
TDav	1
SFO1	500.1315051 MHz
NUC1	1H
P1	10.00 usec
P2	20.00 usec
P28	1000.00 usec
PLW1	19.48699951 W
SFO2	125.7716219 MHz
NUC2	13C
CPDPRG[2	garp
P3	10.00 usec
P4	20.00 usec
PCPD2	70.00 usec
PLW2	85.47299957 W
PLW12	1.74440002 W
GPNAM[1]	SMSQ10.100
GPZ1	80.00 %
GPNAM[2]	SMSQ10.100
GPZ2	20.10 %
GPNAM[3]	SMSQ10.100
GPZ3	11.00 %
GPNAM[4]	SMSQ10.100
GPZ4	-5.00 %
P16	1000.00 usec
P19	600.00 usec

Figure S7: HMBC (4Hz) of compound **1** in 125 & 500 MHz, DMSO-d_6.

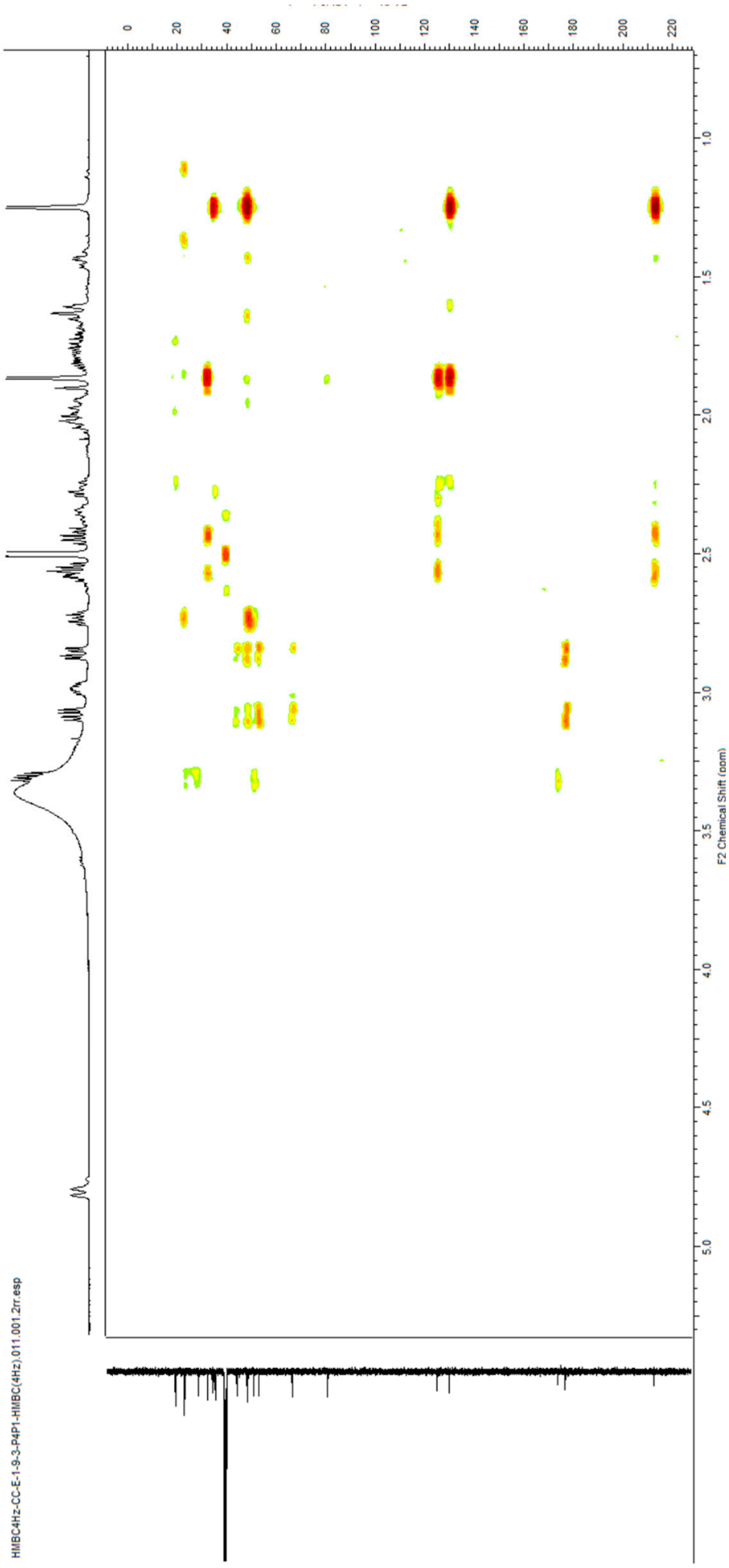

Current Data Parameters
NAME 20210927-Wang-CC-E-1-9-3-P4P1-HMBC(4Hz)

F2 - Acquisition Parameters

Date_	20210927
Time	10.32 h
INSTRUM	spect
PROBHD	Z113652_0220 (
PULPROG	hmbcgplpndqf
TD	512
SOLVENT	DMSO
NS	8
DS	16
SWH	2325.581 Hz
FIDRES	9.084302 Hz
AQ	0.1100800 sec
RG	186.63
DW	215.000 usec
DE	6.50 usec
TE	297.6 K
CNST2	145.0000000
CNST13	4.0000000
D0	0.00000300 sec
D1	1.50000000 sec
D2	0.00344828 sec
D6	0.12500000 sec
D16	0.00020000 sec
IN0	0.00001680 sec
TDav	1
SFO1	500.1315054 MHz
NUC1	1H
P1	10.00 usec
P2	20.00 usec
PLW1	19.48699951 W
SFO2	125.7716219 MHz
NUC2	13C
P3	10.00 usec
PLW2	85.47299957 W
GPNAM[1]	SMSQ10.100
GPZ1	50.00 %
GPNAM[2]	SMSQ10.100
GPZ2	30.00 %
GPNAM[3]	SMSQ10.100
GPZ3	40.10 %
P16	1000.00 usec

Figure S8: HMBC (10Hz) of compound **1** in 125 & 500 MHz, DMSO-d_6.

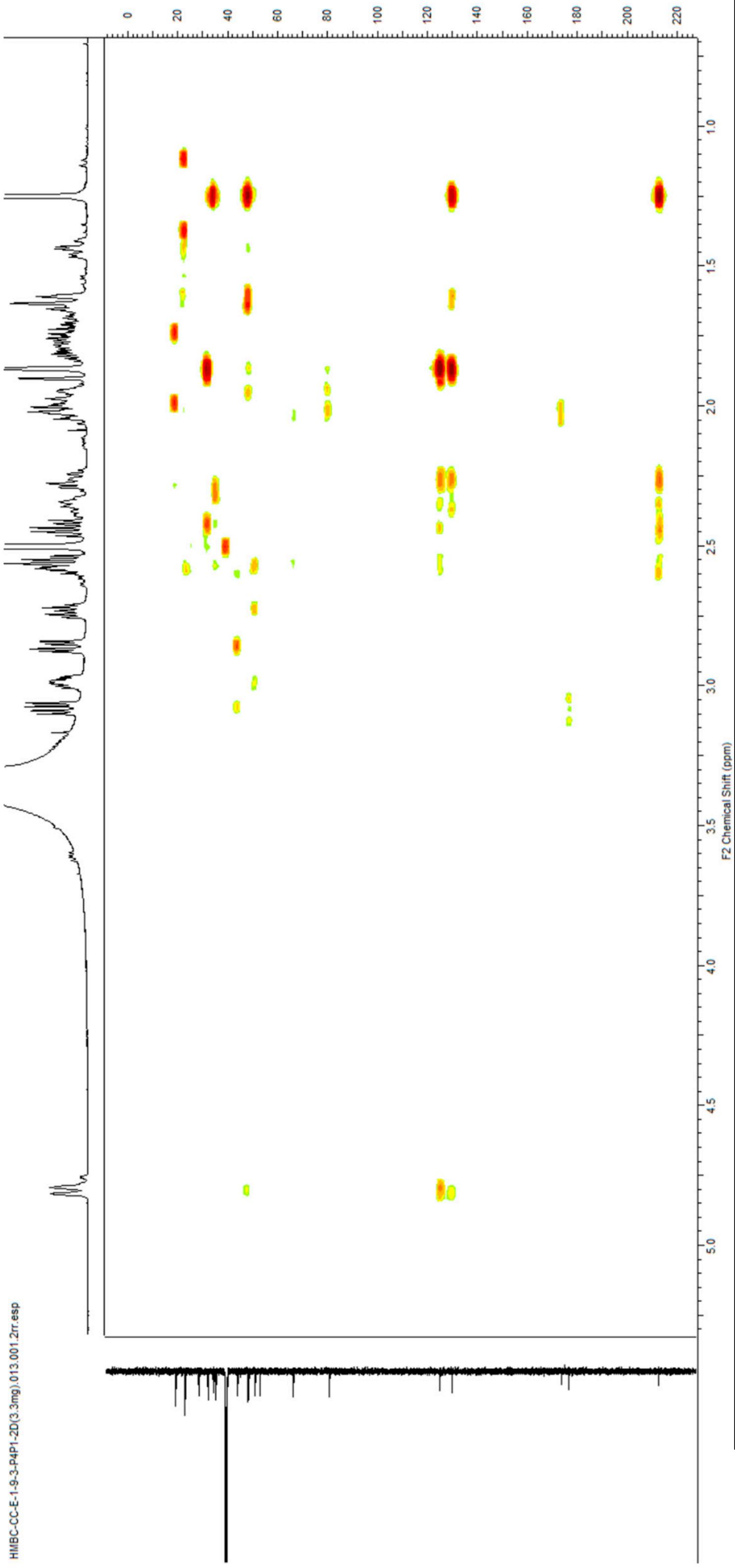

Current Data Parameters
NAME 20210926-Wang-CC-E-1-9-3-P4P1(3.3mg)
F2 - Acquisition Parameters

Date_	20210926
Time	10.41 h
INSTRUM	spect
PROBHD	Z113652_0220 (
PULPROG	hmbcgplpndqf
TD	512
SOLVENT	DMSO
NS	8
DS	16
SWH	2325.581 Hz
FIDRES	9.084302 Hz
AQ	0.1100800 sec
RG	186.63
DW	215.000 usec
DE	6.50 usec
TE	298.0 K
CNST2	145.0000000
CNST13	10.0000000
D0	0.00000300 sec
D1	1.50000000 sec
D2	0.00344828 sec
D6	0.05000000 sec
D16	0.00020000 sec
IN0	0.00001680 sec
TDav	1
SFO1	500.1315051 MHz
NUC1	1H
P1	10.00 usec
P2	20.00 usec
PLW1	19.48699951 W
SFO2	125.7716219 MHz
NUC2	13C
P3	10.00 usec
PLW2	85.47299957 W
GPNAM[1]	SMSQ10.100
GPZ1	50.00 %
GPNAM[2]	SMSQ10.100
GPZ2	30.00 %
GPNAM[3]	SMSQ10.100
GPZ3	40.10 %
P16	1000.00 usec

Figure S9: PS-NOESY of compound **1** in 500 MHz, DMSO-d_6.

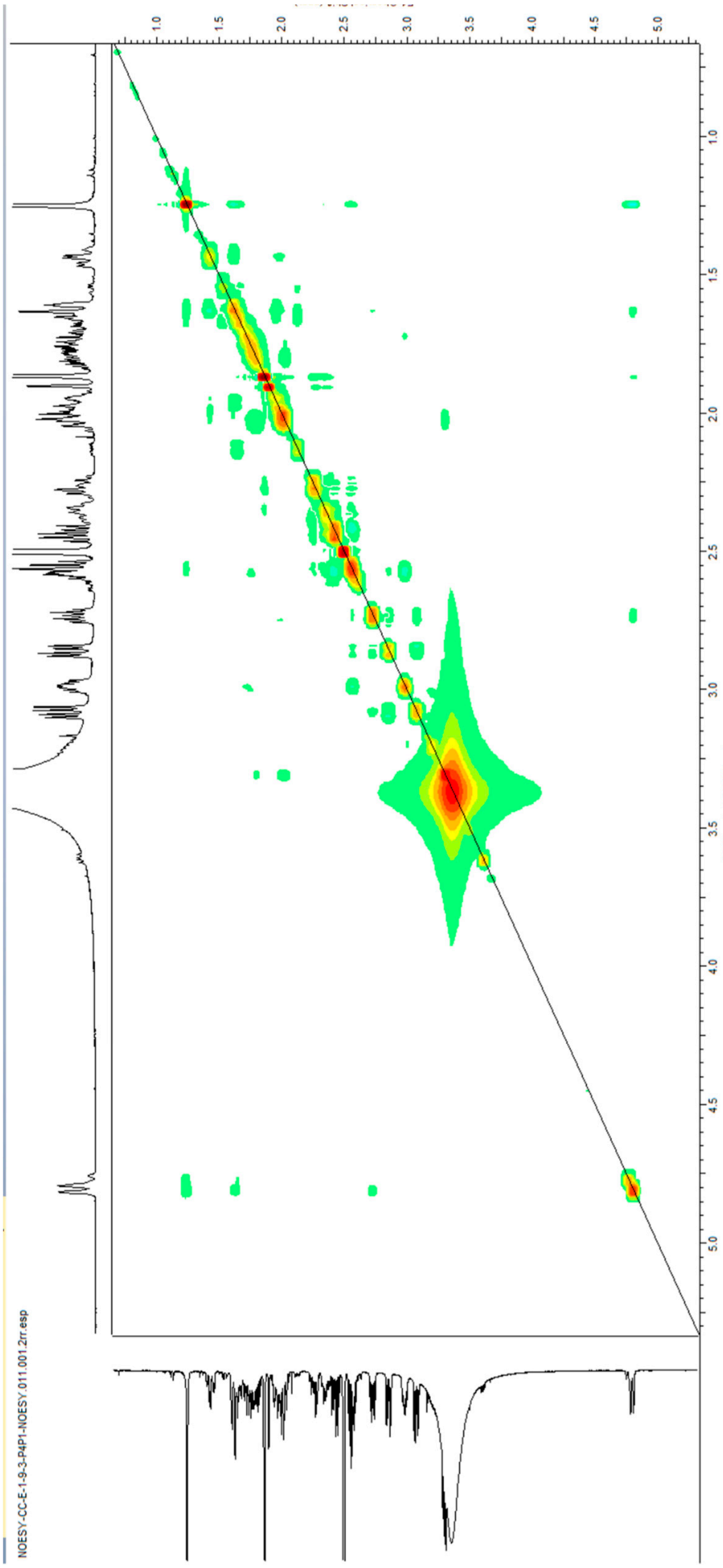

Current Data Parameters

NAME 20210930-Wamg-CC-E-1-9-3-P4P1-NOESY

F2 - Acquisition Parameters

Date_	20210930
Time	10.41 h
INSTRUM	spect
PROBHD	Z113652_0220 (
PULPROG	noesygpphpp
TD	2048
SOLVENT	DMSO
NS	4
DS	32
SWH	2336.448 Hz
FIDRES	2.281688 Hz
AQ	0.4382720 sec
RG	61.94
DW	214.000 usec
DE	8.40 usec
TE	297.8 K
D0	0.00020127 sec
D1	1.69826102 sec
D8	0.80000001 sec
D11	0.03000000 sec
D12	0.00002000 sec
D16	0.00020000 sec
IN0	0.00042800 sec
TDav	1
SFO1	500.1315006 MHz
NUC1	1H
P1	10.00 usec
P2	20.00 usec
P17	2500.00 usec
PLW1	19.48699951 W
PLW10	2.16520000 W
GPNAM[1]	SMSQ10.100
GPZ1	40.00 %
P16	1000.00 usec

Figure S10: HR-ESI-MS of compound **1**.

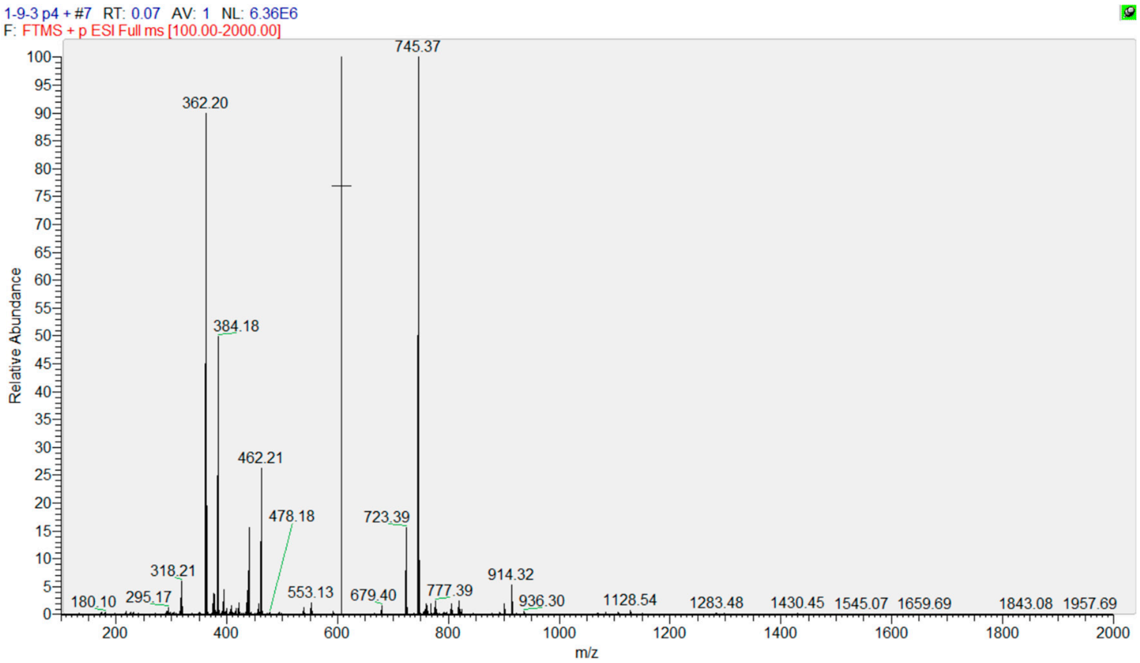

Elemental composition search on mass 362.20

m/z= 357.20-367.20

m/z	Theo. Mass	Delta (ppm)	RDB equiv.	Composition
362.1961	362.1962	-0.15	13.0	$C_{19}H_{22}N_8$
	362.1962	-0.16	7.5	$C_{20}H_{28}O_5N$
	362.1949	3.54	8.0	$C_{18}H_{26}O_4N_4$
	362.1975	-3.86	12.5	$C_{21}H_{24}ON_5$
	362.1980	-5.26	0.0	$C_7H_{26}O_7N_{10}$
	362.1935	7.24	3.0	$C_{17}H_{30}O_8$
	362.1935	7.25	8.5	$C_{16}H_{24}O_3N_7$
	362.1989	-7.56	12.0	$C_{23}H_{26}O_2N_2$
	362.1994	-8.97	-0.5	$C_9H_{28}O_8N_7$
	362.1922	10.94	3.5	$C_{15}H_{28}O_7N_3$

Figure S11: ESI-MS/MS of compound **1**.

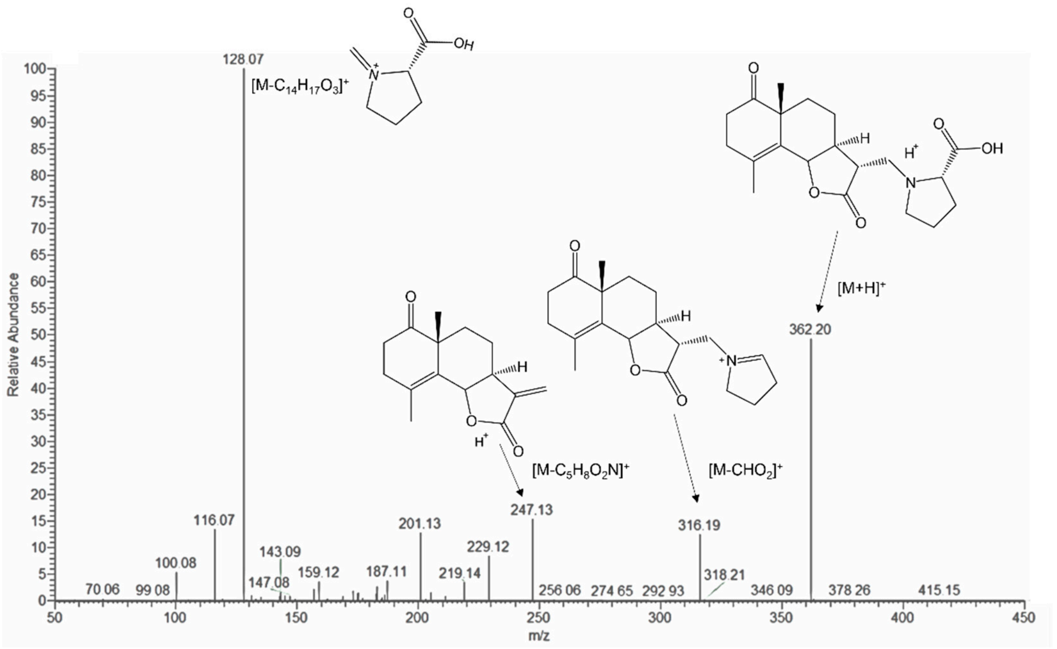

Figure S12: CD spectrum of compound **1**, MeOH.

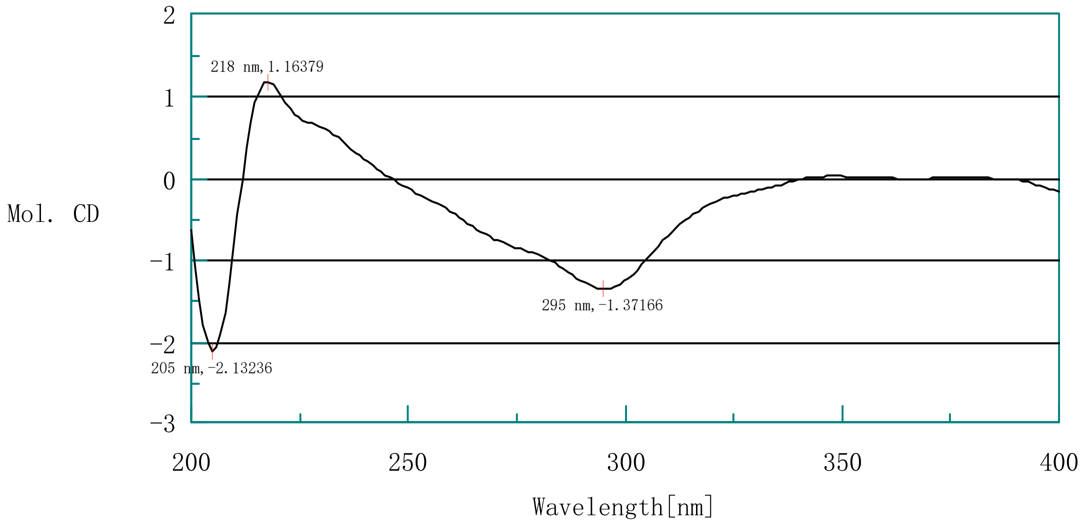

Concentration: 2.75*10^{-5} M

Figure S13: ^{1}H NMR of compound **2** in 500 MHz, DMSO-d_6.

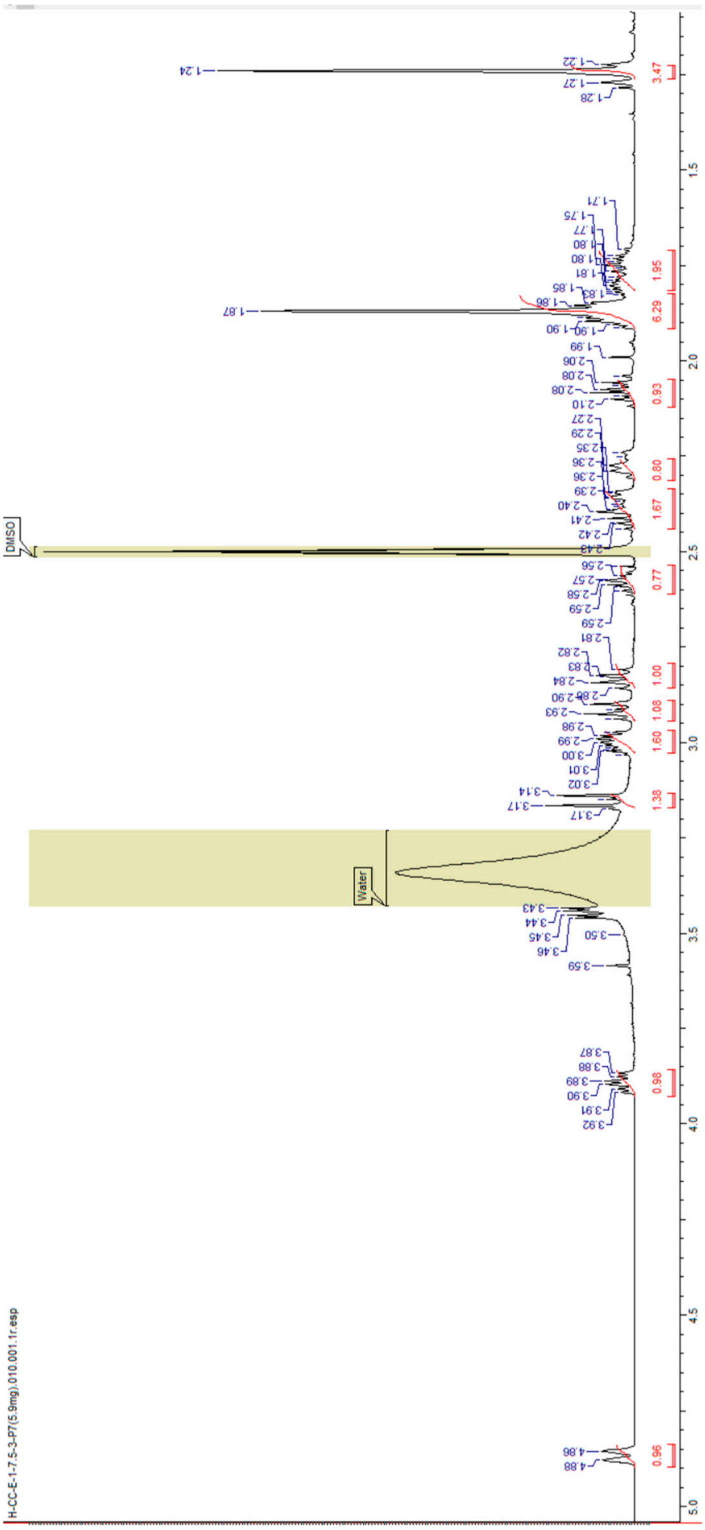

Current Data Parameters	
NAME	20210928-Wang-CC-E-1-7.5-3-P7(5.9mg)
F2 - Acquisition Parameters	
Date_	20210928
Time	13.35 h
INSTRUM	spect
PROBHD	Z113652_0220 (
PULPROG	zg30
TD	65536
SOLVENT	DMSO
NS	16
DS	2
SWH	7500.000 Hz
FIDRES	0.228882 Hz
AQ	4.3690667 sec
RG	110.57
DW	66.667 usec
DE	12.08 usec
TE	297.8 K
D1	1.00000000 sec
TD0	1
SFO1	500.1332508 MHz
NUC1	1H
P0	3.33 usec
P1	10.00 usec
PLW1	19.48699951 W

Figure S14: ^{1}H NMR (4.80 ppm) of compound **2** in 500 MHz, DMSO-d_6.

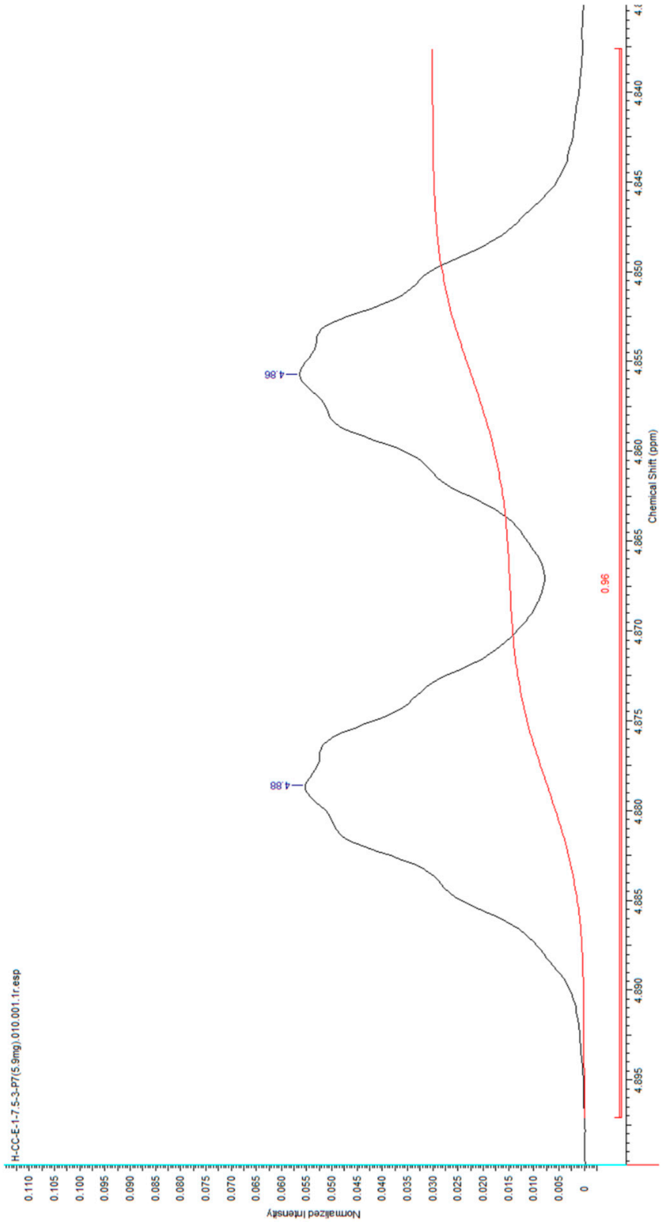

Current Data Parameters	
NAME	20210928-Wang-CC-E-1-7.5-3-P7(5.9mg)
F2 - Acquisition Parameters	
Date_	20210928
Time	13.35 h
INSTRUM	spect
PROBHD	Z113652_0220 (
PULPROG	zg30
TD	65536
SOLVENT	DMSO
NS	16
DS	2
SWH	7500.000 Hz
FIDRES	0.228882 Hz
AQ	4.3690667 sec
RG	110.57
DW	66.667 usec
DE	12.08 usec
TE	297.8 K
D1	1.00000000 sec
TD0	1
SFO1	500.1332508 MHz
NUC1	1H
P0	3.33 usec
P1	10.00 usec
PLW1	19.48699951 W

Figure S15: ^{13}C NMR of compound **2** in 125 MHz, DMSO-d_6.

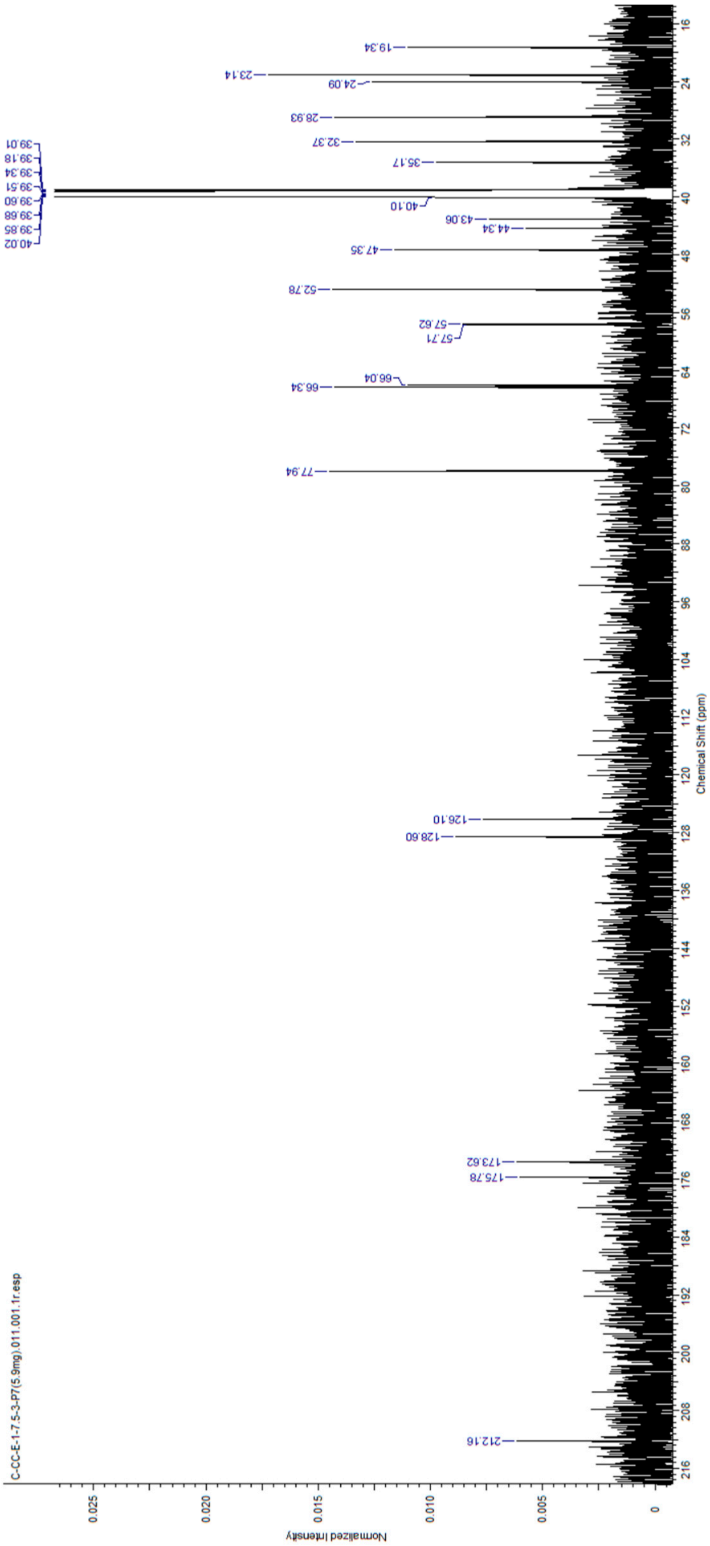

Current Data Parameters

NAME	20210928-Wang-CC-E-1-7.5-3-P7(5.9mg)
F2 - Acquisition Parameters	
Date_	20210928
Time	14.05 h
INSTRUM	spect
PROBHD	Z113652_0220 (
PULPROG	zgpg30
TD	65536
SOLVENT	DMSO
NS	528
DS	4
SWH	29761.904 Hz
FIDRES	0.908261 Hz
AQ	1.1010048 sec
RG	186.63
DW	16.800 usec
DE	6.50 usec
TE	298.8 K
D1	2.00000000 sec
D11	0.03000000 sec
TD0	1
SFO1	125.7716219 MHz
NUC1	13C
P0	3.33 usec
P1	10.00 usec
PLW1	85.47299957 W
SFO2	500.1320005 MHz
NUC2	1H
CPDPRG[2	waltz65
PCPD2	80.00 usec
PLW2	19.48699951 W
PLW12	0.30447999 W
PLW13	0.15315001 W

Figure S16: ^{13}C DEPT NMR of compound **2** in 125 MHz, DMSO-d_6.

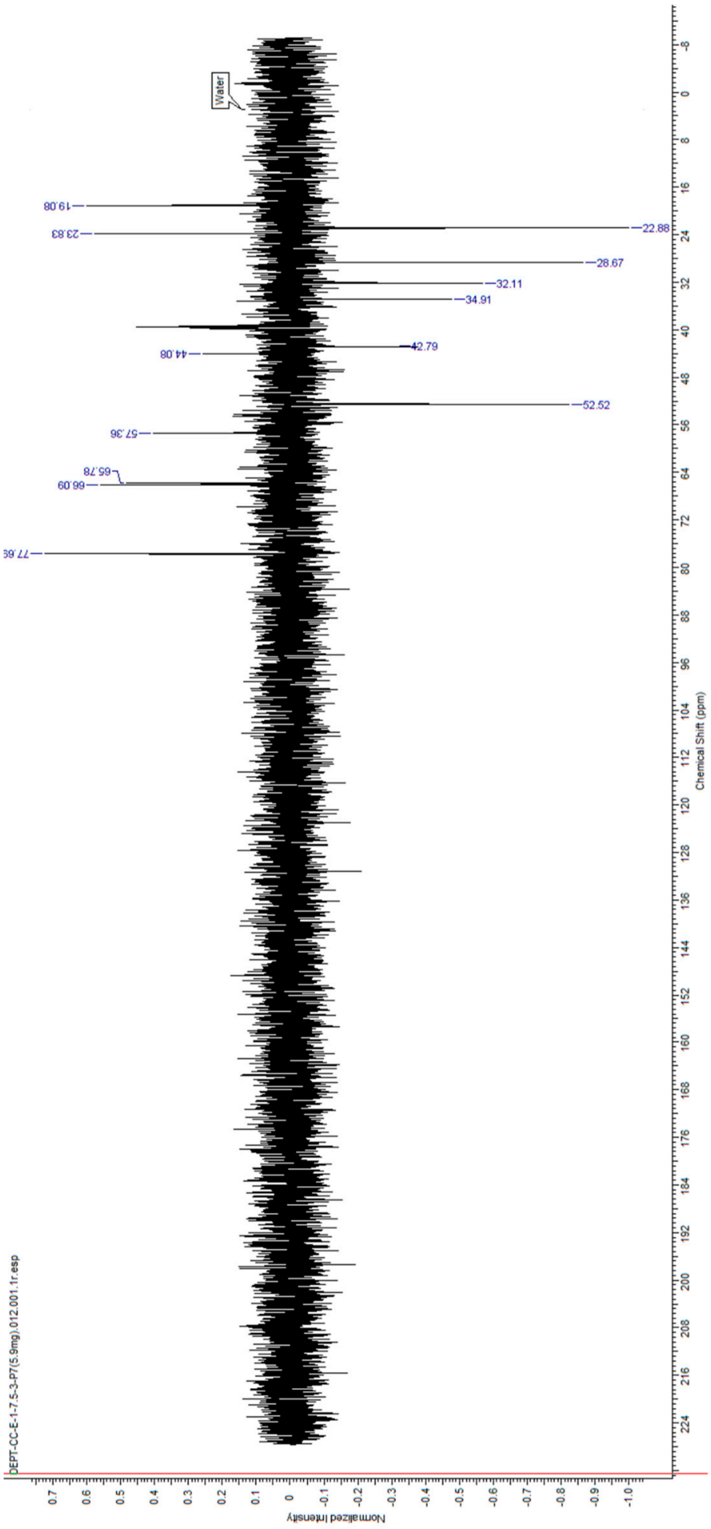

Current Data Parameters	
NAME	20210928-Wang-CC-E-1-7.5-3-P7(5.9mg)
F2 - Acquisition Parameters	
Date_	20210928
Time	14.13 h
INSTRUM	spect
PROBHD	Z113652_0220 (
PULPROG	deptsp135
TD	65536
SOLVENT	DMSO
NS	128
DS	8
SWH	29761.904 Hz
FIDRES	0.908261 Hz
AQ	1.1010048 sec
RG	186.63
DW	16.800 usec
DE	6.50 usec
TE	298.4 K
CNST2	145.0000000
D1	2.00000000 sec
D2	0.00344828 sec
D12	0.00002000 sec
TD0	1
SFO1	125.7716219 MHz
NUC1	13C
P1	10.00 usec
P13	2000.00 usec
PLW0	0 W
PLW1	85.47299957 W
SPNAM[5]	Crp60comp.4
SPOAL5	0.500
SPOFFS5	0 Hz
SPW5	13.05900002 W
SFO2	500.1320005 MHz
NUC2	1H
CPDPRG[2	waltz65
P3	10.00 usec
P4	20.00 usec
PCPD2	80.00 usec
PLW2	19.48699951 W
PLW12	0.30447999 W

Figure S17: COSY of compound **2** in 500 MHz, DMSO-d_6.

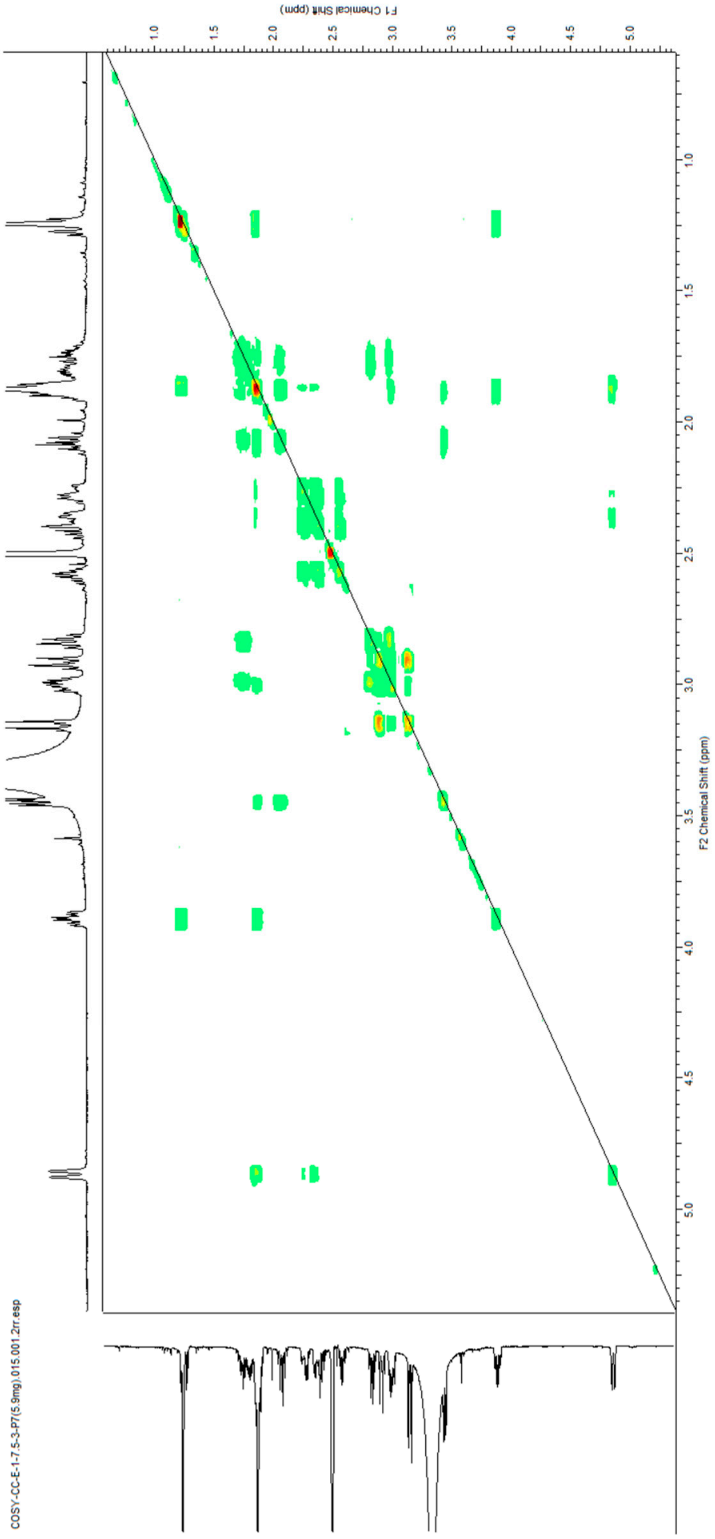

Current Data Parameters	
NAME	20210928-Wang-CC-E-1-7.5-3-P7(5.9mg)
F2 - Acquisition Parameters	
Date_	20210928
Time	14.33 h
INSTRUM	spect
PROBHD	Z113652_0220 (
PULPROG	cosygpppqf
TD	2048
SOLVENT	DMSO
NS	4
DS	16
SWH	2403.846 Hz
FIDRES	2.347506 Hz
AQ	0.4259840 sec
RG	61.94
DW	208.000 usec
DE	6.50 usec
TE	298.4 K
D0	0.00000300 sec
D1	1.71054900 sec
D11	0.03000000 sec
D12	0.00002000 sec
D13	0.00000400 sec
D16	0.00020000 sec
IN0	0.00041600 sec
TDav	1
SFO1	500.1315036 MHz
NUC1	1H
P0	10.00 usec
P1	10.00 usec
P17	2500.00 usec
PLW1	19.48699951 W
PLW10	2.16520000 W
GPNAM[1]	SMSQ10.100
GPZ1	10.00 %
P16	1000.00 usec

Figure S18: HSQC of compound **2** in 125 & 500 MHz, DMSO-d_6.

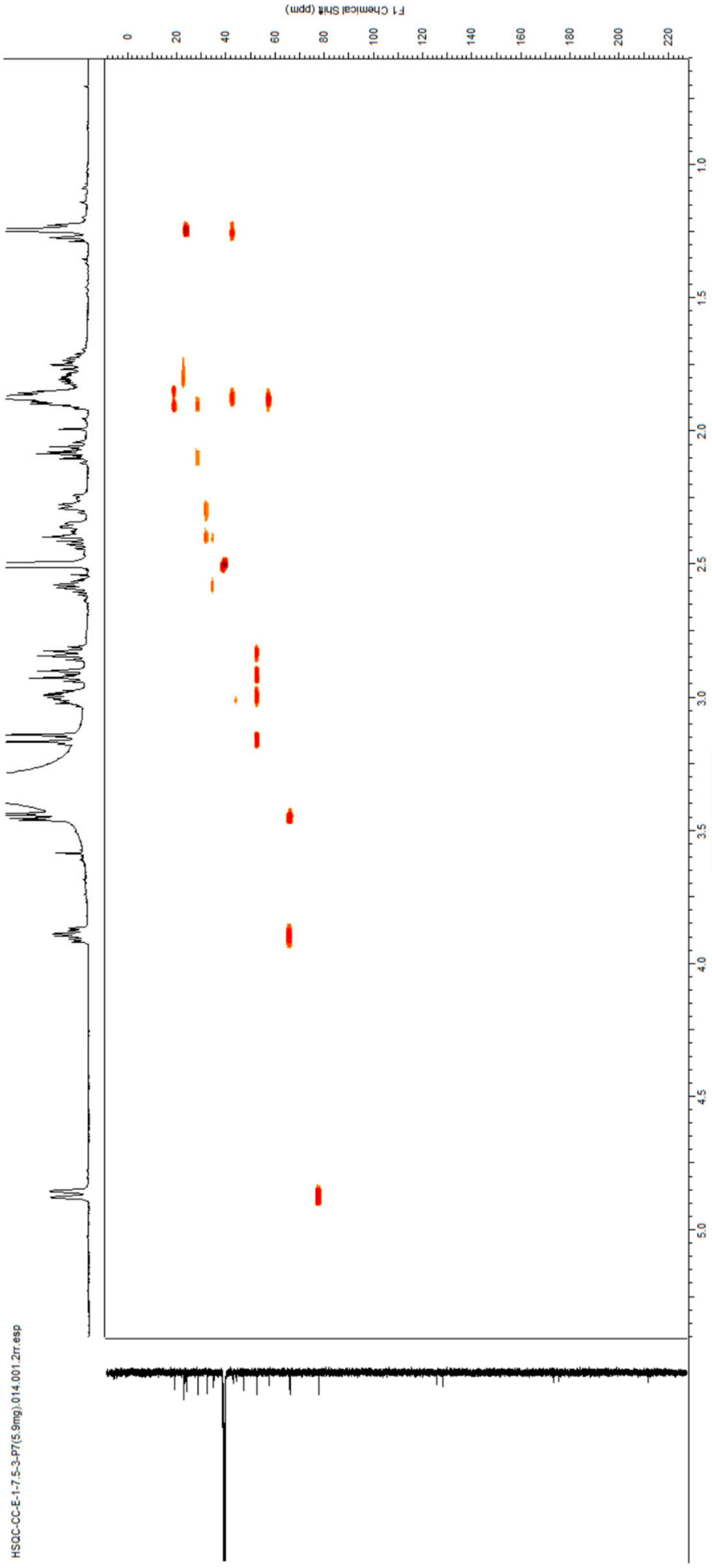

Current Data Parameters

NAME	20210928-Wang-CC-E-1-7.5-3-P7(5.9mg)
F2 - Acquisition Parameters	
Date_	20210928
Time	14.17 h
INSTRUM	spect
PROBHD	Z113652_0220 (
PULPROG	hsqcetgpsi2
TD	1024
SOLVENT	DMSO
NS	2
DS	16
SWH	2403.846 Hz
FIDRES	4.695012 Hz
AQ	0.2129920 sec
RG	186.63
DW	208.000 usec
DE	19.81 usec
TE	298.5 K
CNST2	145.0000000
D0	0.00000300 sec
D1	1.50000000 sec
D4	0.00172414 sec
D11	0.03000000 sec
D16	0.00020000 sec
D24	0.00086207 sec
IN0	0.00001680 sec
TDav	1
SFO1	500.1315036 MHz
NUC1	1H
P1	10.00 usec
P2	20.00 usec
P28	1000.00 usec
PLW1	19.48699951 W
SFO2	125.7716219 MHz
NUC2	13C
CPDPRG[2	garp
P3	10.00 usec
P4	20.00 usec
PCPD2	70.00 usec
PLW2	85.47299957 W
PLW12	1.74440002 W
GPNAM[1]	SMSQ10.100
GPZ1	80.00 %
GPNAM[2]	SMSQ10.100
GPZ2	20.10 %
GPNAM[3]	SMSQ10.100
GPZ3	11.00 %
GPNAM[4]	SMSQ10.100
GPZ4	-5.00 %
P16	1000.00 usec
P19	600.00 usec

Figure S19: HMBC (4Hz) of compound **2** in 125 & 500 MHz, DMSO-d_6.

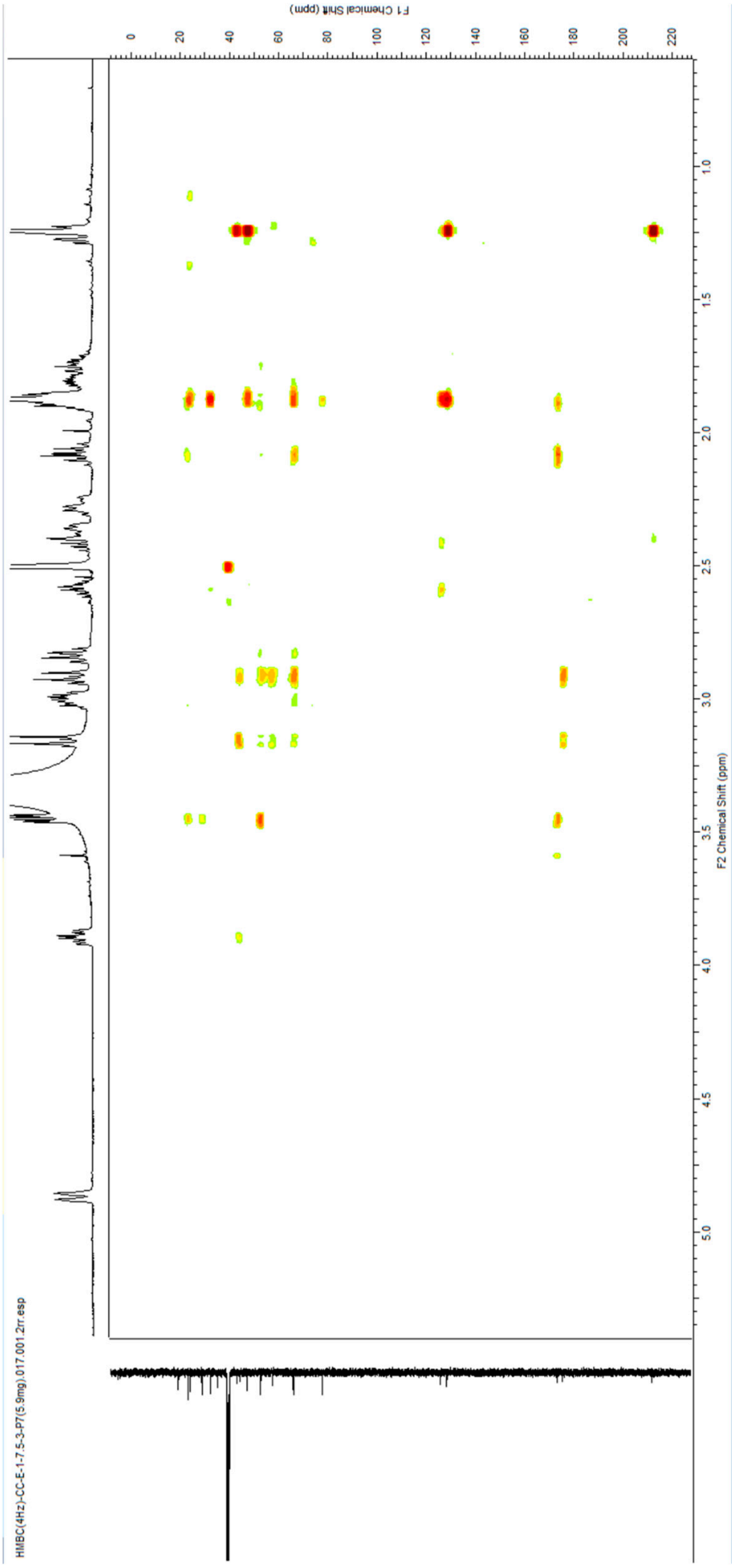

Current Data Parameters	
NAME	20210928-Wang-CC-E-1-7.5-3-P7(5.9mg)
EXPNO	17
PROCNO	1
F2 - Acquisition Parameters	
Date_	20210928
Time	15.10 h
INSTRUM	spect
PROBHD	Z113652_0220 (
PULPROG	hmbcgplpndqf
TD	1024
SOLVENT	DMSO
NS	4
DS	16
SWH	2403.846 Hz
FIDRES	4.695012 Hz
AQ	0.2129920 sec
RG	186.63
DW	208.000 usec
DE	6.50 usec
TE	297.8 K
CNST2	145.0000000
CNST13	4.0000000
D0	0.00000300 sec
D1	1.50000000 sec
D2	0.00344828 sec
D6	0.12500000 sec
D16	0.00020000 sec
IN0	0.00001680 sec
TDav	1
SFO1	500.1315036 MHz
NUC1	1H
P1	10.00 usec
P2	20.00 usec
PLW1	19.48699951 W
SFO2	125.7716219 MHz
NUC2	13C
P3	10.00 usec
PLW2	85.47299957 W
GPNAM[1]	SMSQ10.100
GPZ1	50.00 %
GPNAM[2]	SMSQ10.100
GPZ2	30.00 %
GPNAM[3]	SMSQ10.100
GPZ3	40.10 %
P16	1000.00 usec

Figure S20: HMBC (10Hz) of compound **2** in 125 & 500 MHz, DMSO-d_6.

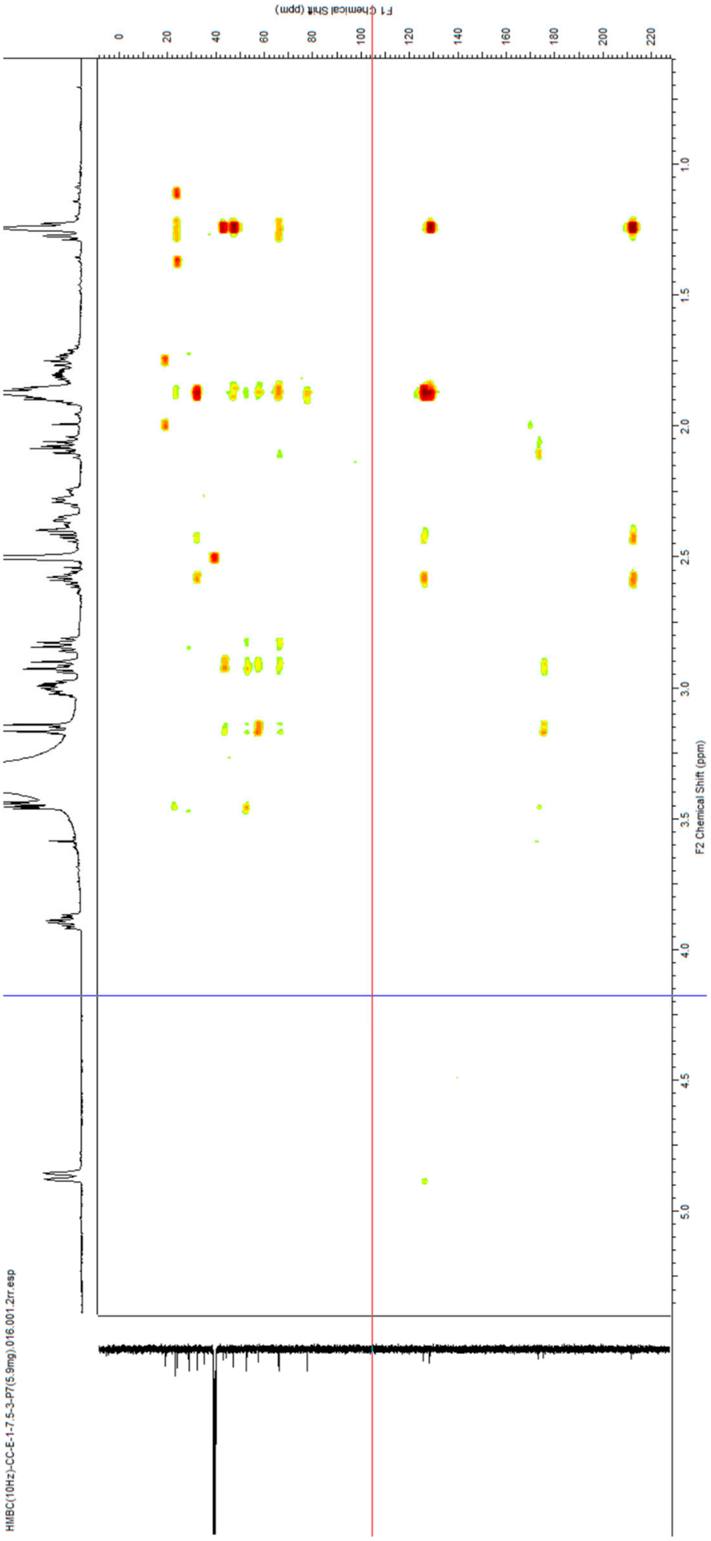

Current Data Parameters

NAME	20210928-Wang-CC-E-1-7.5-3-P7(5.9mg)
EXPNO	16
PROCNO	1

F2 - Acquisition Parameters

Date_	20210928
Time	14.54 h
INSTRUM	spect
PROBHD	Z113652_0220 (
PULPROG	hmbcgplpndqf
TD	1024
SOLVENT	DMSO
NS	4
DS	16
SWH	2403.846 Hz
FIDRES	4.695012 Hz
AQ	0.2129920 sec
RG	186.63
DW	208.000 usec
DE	6.50 usec
TE	297.9 K
CNST2	145.0000000
CNST13	10.0000000
D0	0.00000300 sec
D1	1.50000000 sec
D2	0.00344828 sec
D6	0.05000000 sec
D16	0.00020000 sec
IN0	0.00001680 sec
TDav	1
SFO1	500.1315036 MHz
NUC1	1H
P1	10.00 usec
P2	20.00 usec
PLW1	19.48699951 W
SFO2	125.7716219 MHz
NUC2	13C
P3	10.00 usec
PLW2	85.47299957 W
GPNAM[1]	SMSQ10.100
GPZ1	50.00 %
GPNAM[2]	SMSQ10.100
GPZ2	30.00 %
GPNAM[3]	SMSQ10.100
GPZ3	40.10 %
P16	1000.00 usec

Figure S21: PS-NOESY of compound **2** in 500 MHz, DMSO-d_6.

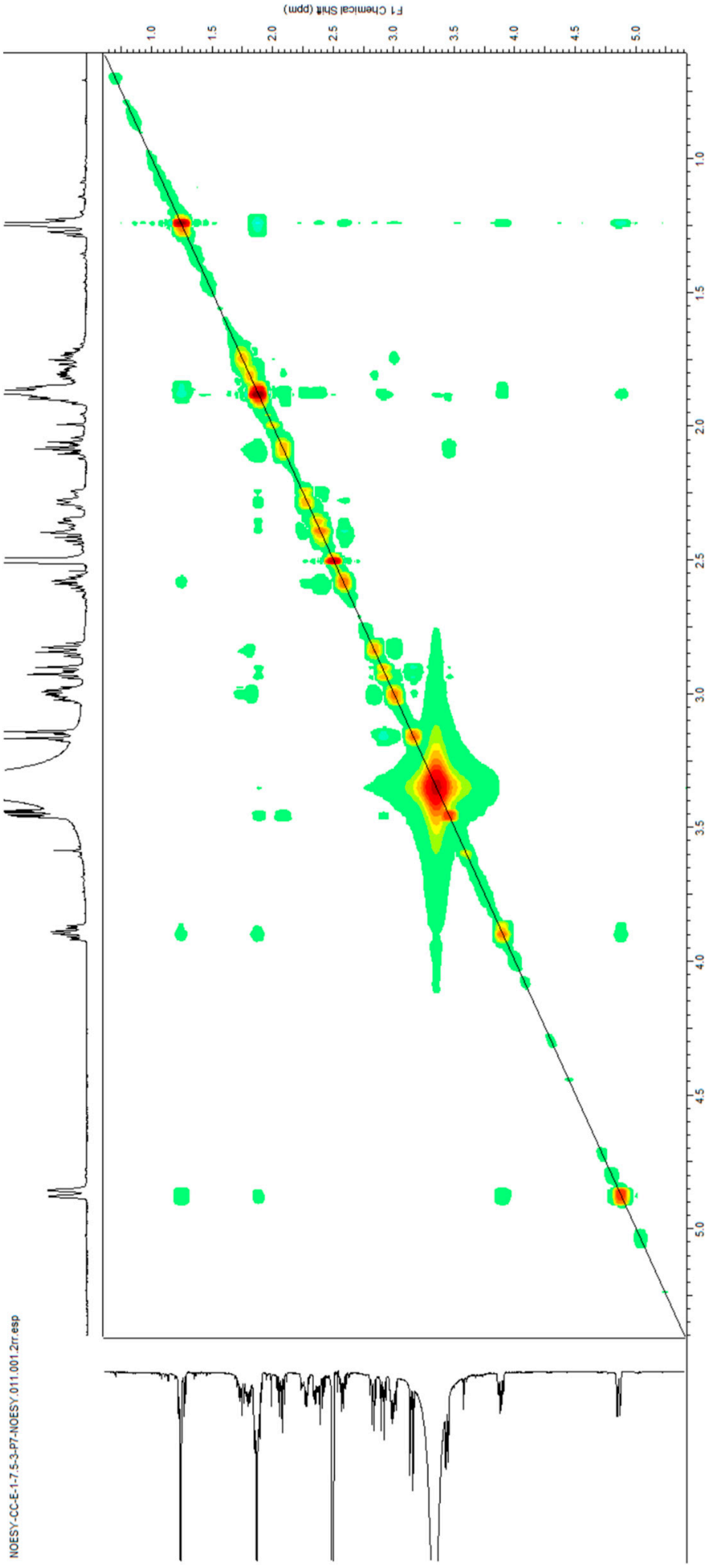

Current Data Parameters	
NAME	20210930-Wang-CC-E-1-7.5-3-P7-NOESY
EXPNO	11
PROCNO	1
F2 - Acquisition Parameters	
Date_	20210930
Time	11.31 h
INSTRUM	spect
PROBHD	Z113652_0220 (
PULPROG	noesygpphpp
TD	2048
SOLVENT	DMSO
NS	4
DS	32
SWH	2403.846 Hz
FIDRES	2.347506 Hz
AQ	0.4259840 sec
RG	61.94
DW	208.000 usec
DE	8.03 usec
TE	297.9 K
D0	0.00019527 sec
D1	1.71054900 sec
D8	0.80000001 sec
D11	0.03000000 sec
D12	0.00002000 sec
D16	0.00020000 sec
IN0	0.00041600 sec
TDav	1
SFO1	500.1315035 MHz
NUC1	1H
P1	10.00 usec
P2	20.00 usec
P17	2500.00 usec
PLW1	19.48699951 W
PLW10	2.16520000 W
GPNAM[1]	SMSQ10.100
GPZ1	40.00 %
P16	1000.00 usec

Figure S22: HR-ESI-MS of compound **2**.

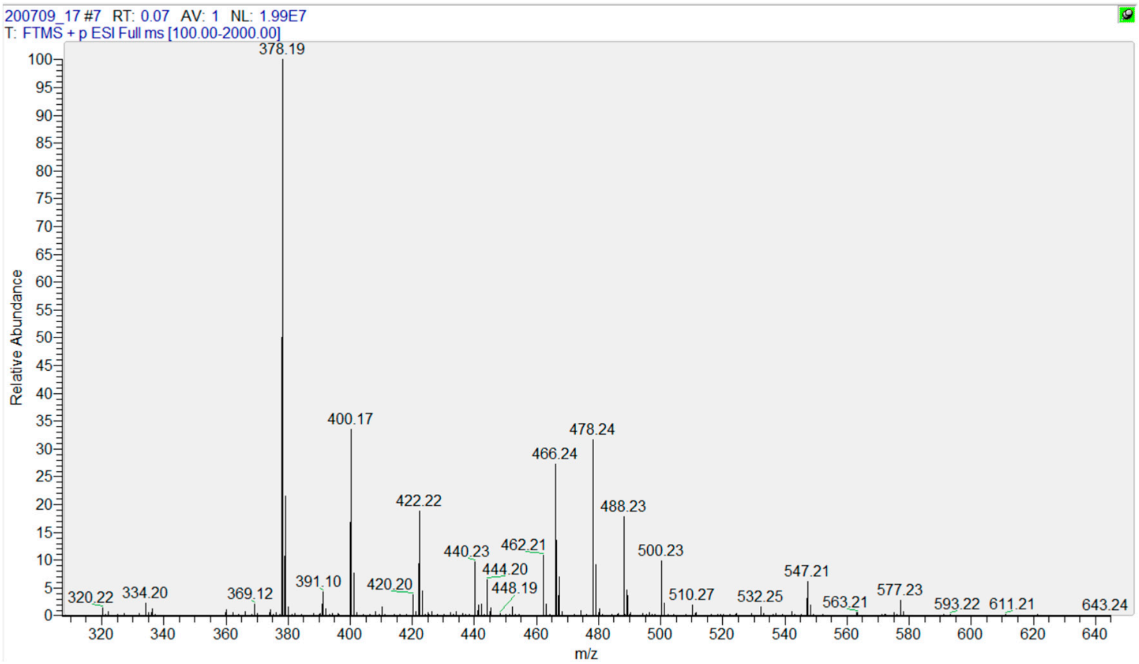

Elemental composition search on mass 378.19

m/z= 373.19-383.19

m/z	Theo. Mass	Delta (ppm)	RDB equiv.	Composition
378.1912	378.1911	0.28	7.5	$C_{20}H_{28}O_6N$
	378.1911	0.29	13.0	$C_{19}H_{22}ON_8$
	378.1925	-3.26	12.5	$C_{21}H_{24}O_2N_5$
	378.1898	3.83	8.0	$C_{18}H_{26}O_5N_4$
	378.1930	-4.60	0.0	$C_7H_{26}O_8N_{10}$
	378.1938	-6.81	12.0	$C_{23}H_{26}O_3N_2$
	378.1884	7.37	3.0	$C_{17}H_{30}O_9$
	378.1884	7.38	8.5	$C_{16}H_{24}O_4N_7$
	378.1943	-8.15	-0.5	$C_9H_{28}O_9N_7$
	378.1871	10.92	3.5	$C_{15}H_{28}O_8N_3$

Figure S23: ESI-MS/MS of compound **2**.

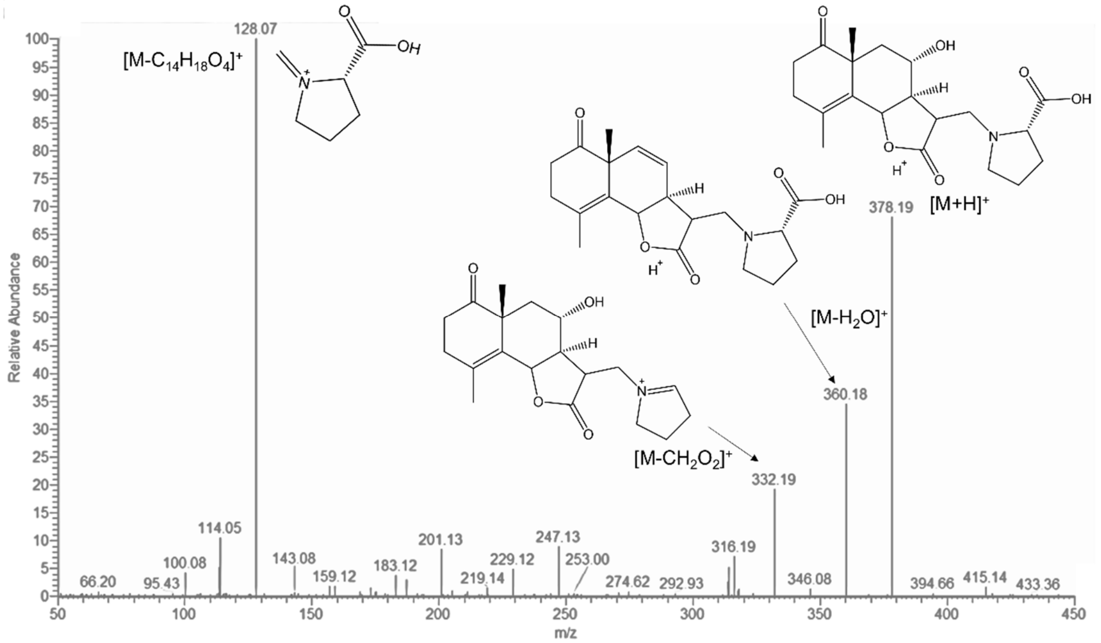

Figure S24: CD spectrum of compound **2**, MeOH.

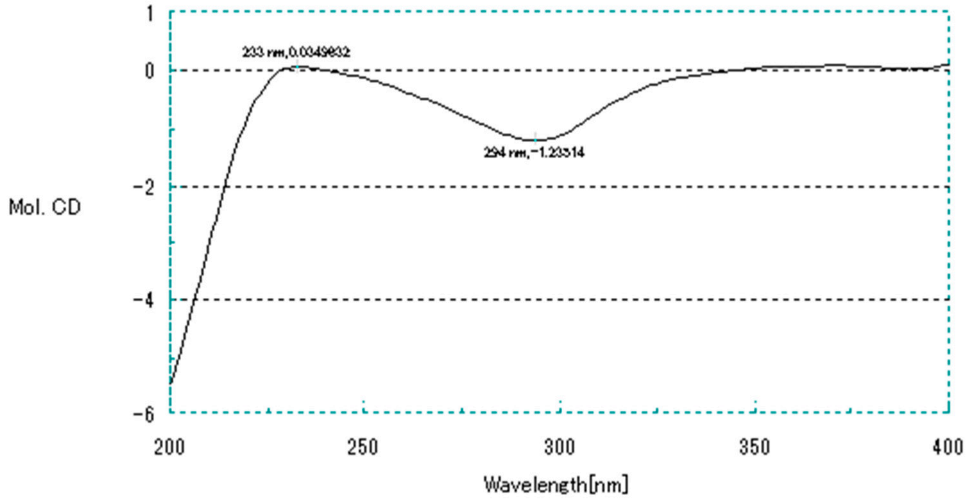

Concentration: $3*10^{-5}$ M

Figure S25: ^{1}H NMR of compound **3** in 500 MHz, Pyridine-d_5.

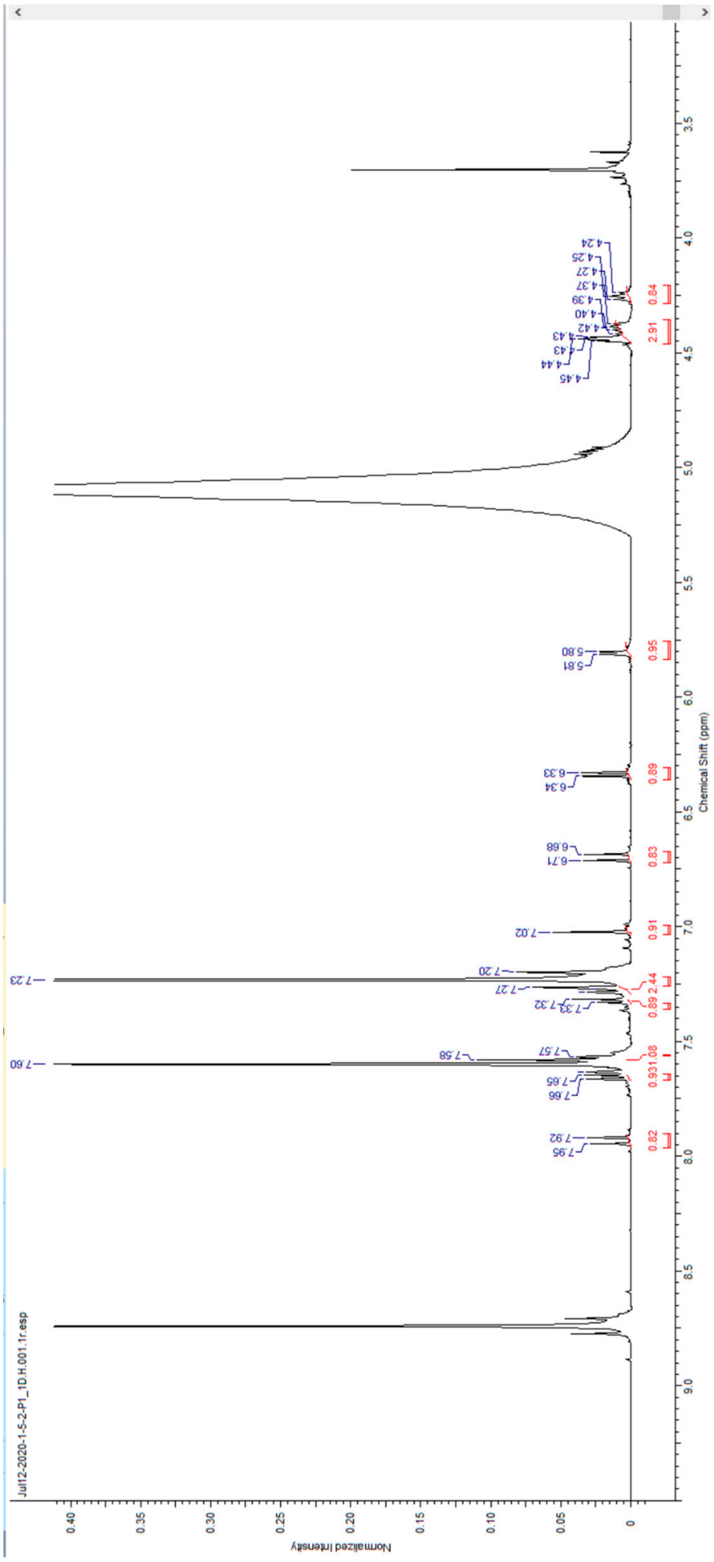

Date_	20200712
Time	11.14
INSTRUM	spect
PROBHD	5 mm PABBO BB-
PULPROG	zg30
TD	65536
SOLVENT	Pyr
NS	16
DS	2
SWH	12335.526 Hz
FIDRES	0.188225 Hz
AQ	2.6564426 sec
RG	57
DW	40.533 usec
DE	6.50 usec
TE	298.5 K
D1	1.00000000 sec
TD0	1

Figure S26: ^{13}C NMR of compound **3** in 125 MHz, Pyridine-d_5.

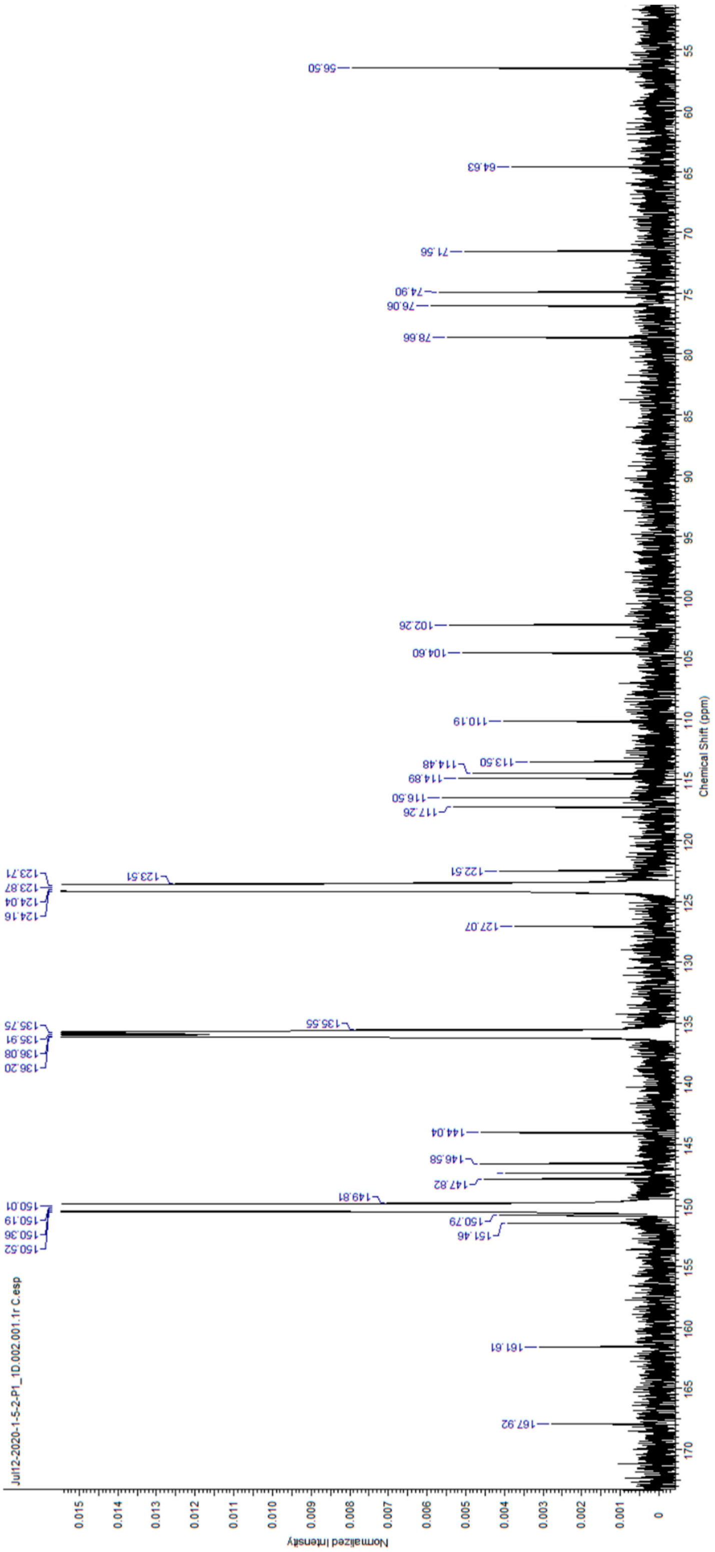

NAME	Jul12-2020-kawakami
EXPNO	2
PROCNO	1
Date_	20200712
Time	14.38
INSTRUM	spect
PROBHD	5 mm PABBO BB-
PULPROG	zgpg30
TD	65536
SOLVENT	Pyr
NS	4096
DS	4
SWH	36057.691 Hz
FIDRES	0.550197 Hz
AQ	0.9088159 sec
RG	2050
DW	13.867 usec
DE	6.50 usec
TE	298.4 K
D1	2.00000000 sec
D11	0.03000000 sec
TD0	1

Figure S27: ^{13}C DEPT NMR of compound **3** in 125 MHz, Pyridine-*d*$_5$.

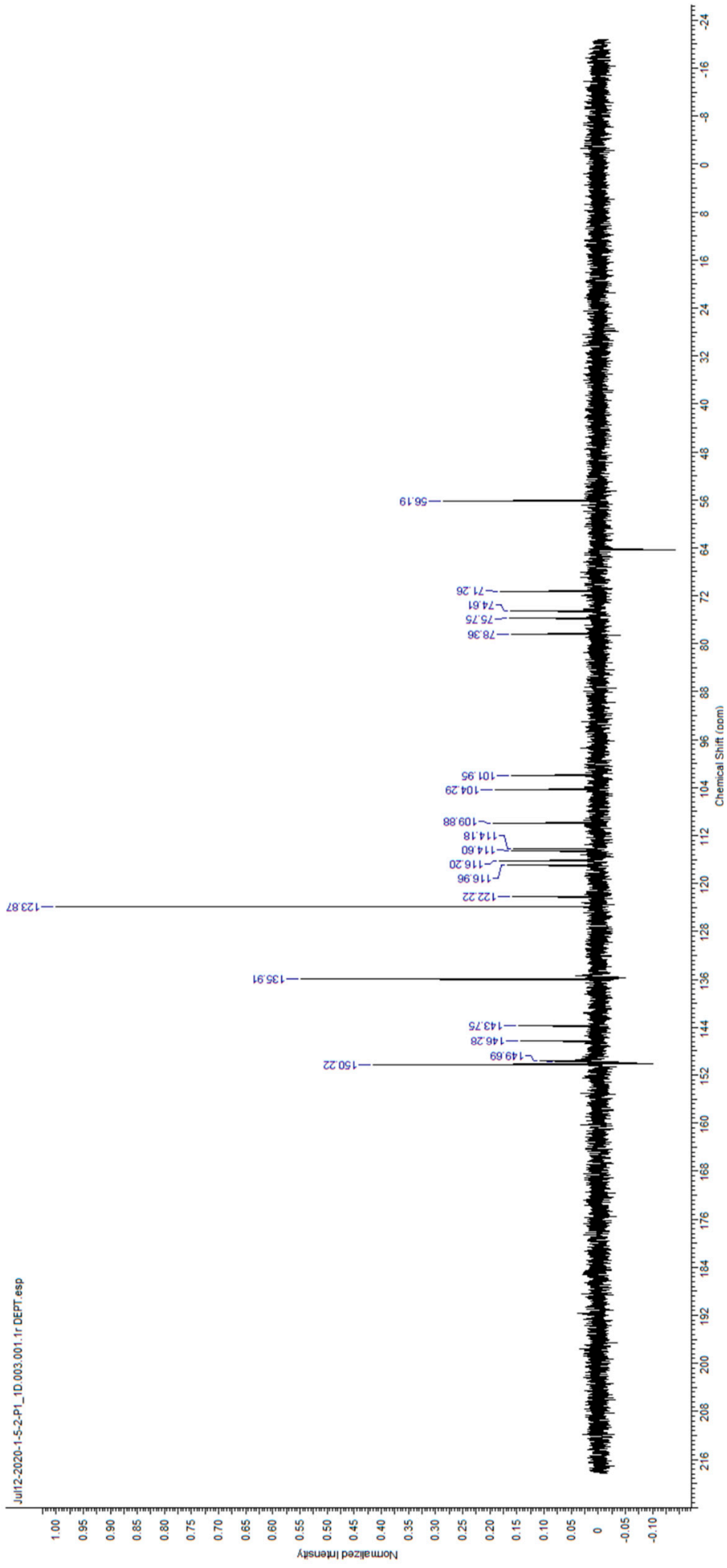

NAME	Jul12-2020-kawakami
EXPNO	3
PROCNO	1
Date_	20200712
Time	15.30
INSTRUM	spect
PROBHD	5 mm PABBO BB-
PULPROG	dept135
TD	65536
SOLVENT	Pyr
NS	1024
DS	4
SWH	36057.691 Hz
FIDRES	0.550197 Hz
AQ	0.9088159 sec
RG	2050
DW	13.867 usec
DE	6.50 usec
TE	298.3 K
CNST2	145.0000000
D1	2.00000000 sec
D2	0.00344828 sec
D12	0.00002000 s

Figure S28: COSY of compound **3** in 500 MHz, Pyridine-d_5.

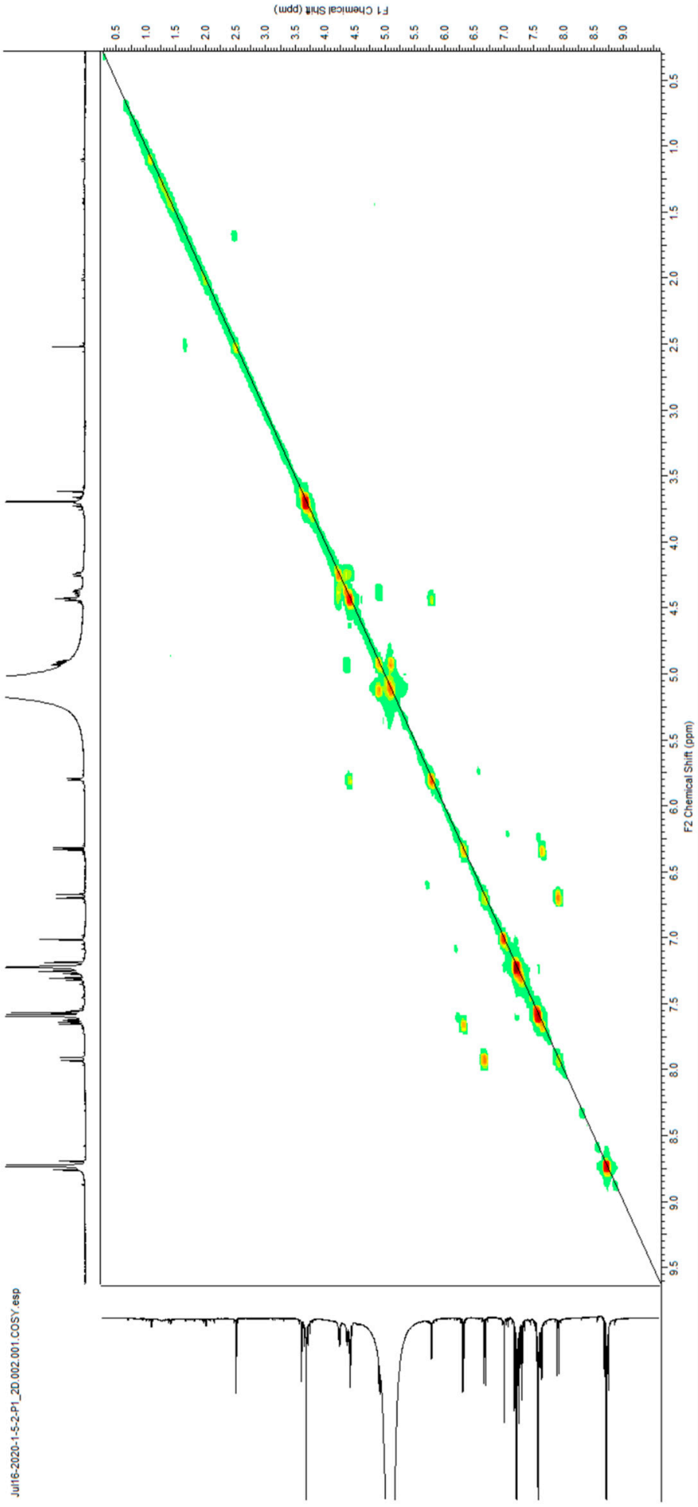

NAME	Jul16-2020-kawakami
EXPNO	2
PROCNO	1
Date_	20200716
Time	19.00
INSTRUM	spect
PROBHD	5 mm PABBO BB-
PULPROG	cosygpqf
TD	2048
SOLVENT	Pyr
NS	16
DS	8
SWH	5617.978 Hz
FIDRES	2.743153 Hz
AQ	0.1823220 sec
RG	50.8
DW	89.000 usec
DE	6.50 usec
TE	298.3 K
D0	0.00000300 sec
D1	1.43241501 sec
D13	0.00000400 sec
D16	0.00020000 sec

Figure S29: HSQC of compound **3** in 125 & 500 MHz, Pyridine-d_5.

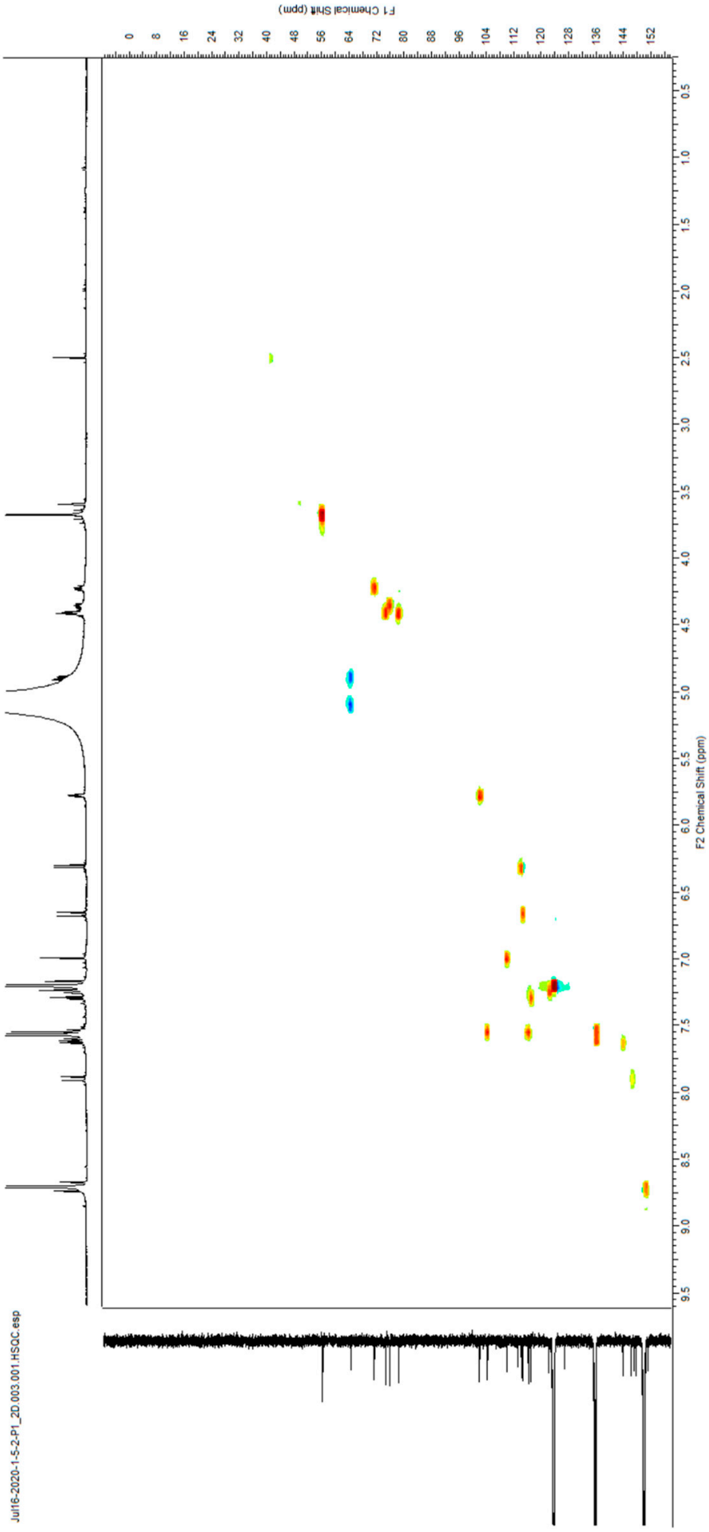

NAME	Jul16-2020-kawakami
EXPNO	3
PROCNO	1
Date_	20200716
Time	19.57
INSTRUM	spect
PROBHD	5 mm PABBO BB-
PULPROG	hsqcedetgp
TD	1024
SOLVENT	Pyr
NS	16
DS	16
SWH	5617.978 Hz
FIDRES	5.486306 Hz
AQ	0.0911860 sec
RG	2050
DW	89.000 usec
DE	6.50 usec
TE	298.8 K
CNST2	145.0000000
D0	0.00000300 sec
D1	1.47276199 sec
D4	0.00172414 sec
D11	0.03000000 sec
D13	0.00000400 sec
D16	0.00020000 sec
D21	0.00345000 sec
IN0	0.00002000 sec
ZGOPTNS	

Figure S30: HMBC (10Hz) of compound **3** in 125 & 500 MHz, Pyridine-d_5.

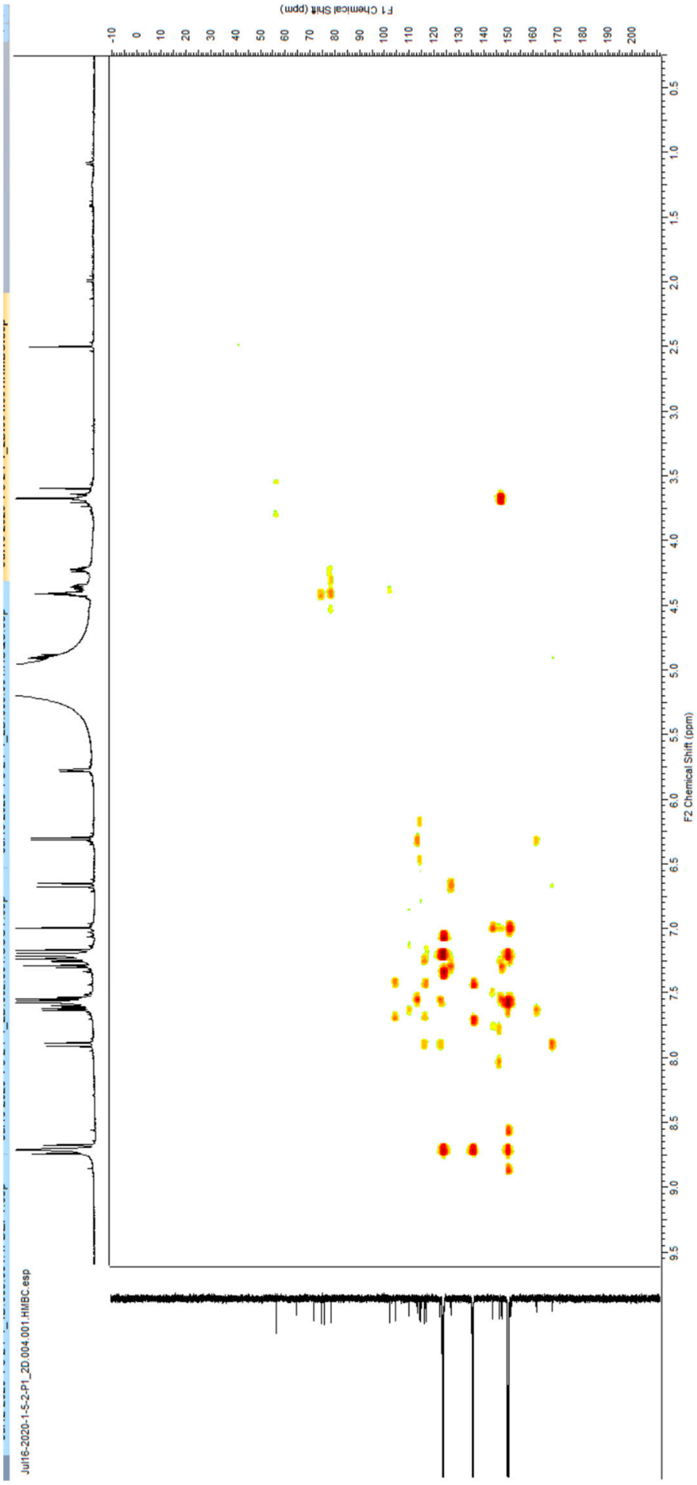

NAME	Jul16-2020-kawakami
EXPNO	4
PROCNO	1
Date_	20200716
Time	21.51
INSTRUM	spect
PROBHD	5 mm PABBO BB-
PULPROG	hmbcgpndqf
TD	4096
SOLVENT	Pyr
NS	144
DS	16
SWH	5617.978 Hz
FIDRES	1.371577 Hz
AQ	0.3645940 sec
RG	2050
DW	89.000 usec
DE	6.50 usec
TE	298.5 K
CNST13	8.0000000
D0	0.00000300 sec
D1	1.39104605 sec
D6	0.06250000 sec
D16	0.00020000 sec
IN0	0.00001490 sec

Figure S31: HR-ESI-MS of compound **3**.

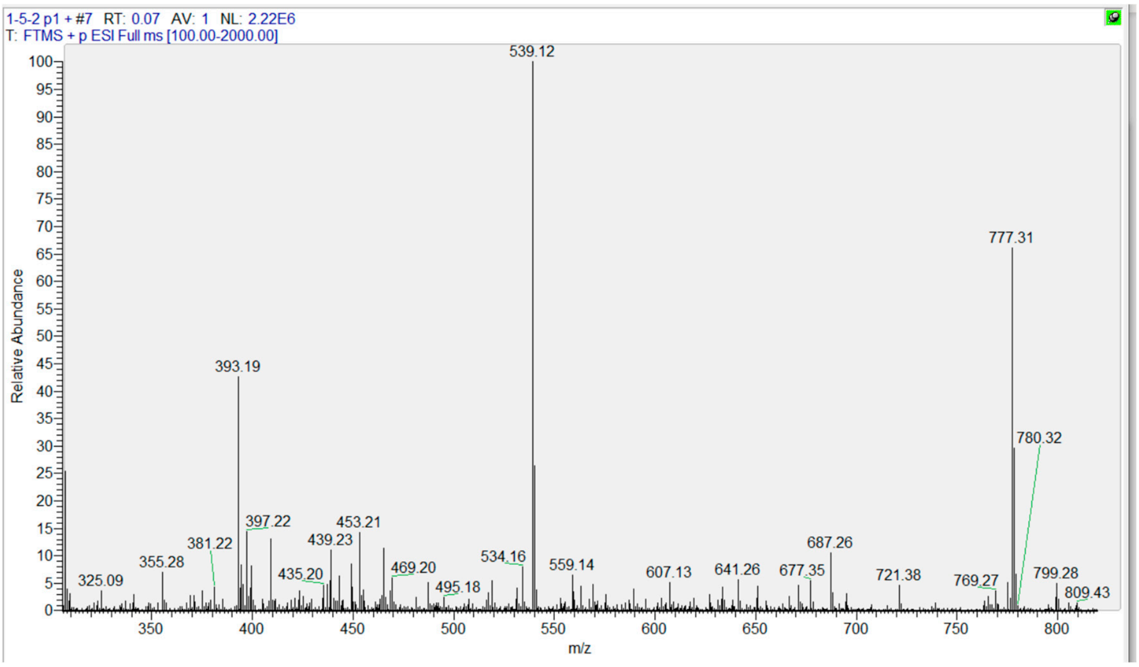

Elemental composition search on mass 539.12

m/z= 534.12-544.12

m/z	Theo. Mass	Delta (ppm)	RDB equiv.	Composition
539.1163	539.1160	0.51	13.5	$C_{25}H_{24}O_{12}Na$
	539.1160	0.52	19.0	$C_{24}H_{18}O_{7}N_{7}Na$
	539.1157	1.03	17.5	$C_{23}H_{19}O_{10}N_{6}$
	539.1171	-1.46	22.5	$C_{24}H_{15}O_{6}N_{10}$
	539.1171	-1.47	17.0	$C_{25}H_{21}O_{11}N_{3}$
	539.1173	-1.97	18.5	$C_{26}H_{20}O_{8}N_{4}Na$
	539.1178	-2.92	6.0	$C_{12}H_{22}O_{14}N_{9}Na$
	539.1147	3.00	14.0	$C_{23}H_{22}O_{11}N_{3}Na$
	539.1146	3.01	19.5	$C_{22}H_{16}O_{6}N_{10}Na$
	539.1144	3.51	12.5	$C_{22}H_{23}O_{14}N_{2}$

Figure S32: ESI-MS/MS of compound **3**.

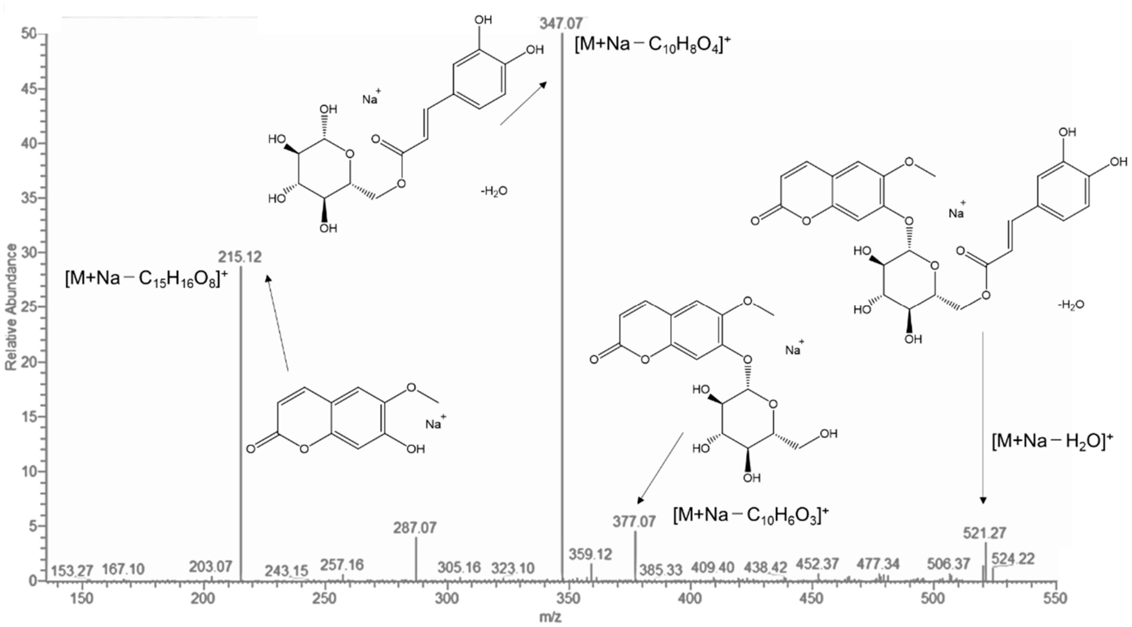

Table S1: Comparison of NMR data of **3** with related compounds **A** and **B**.

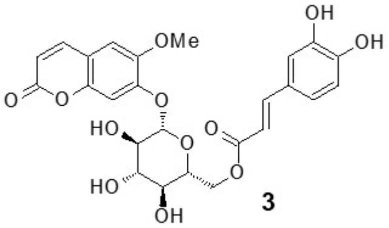

3

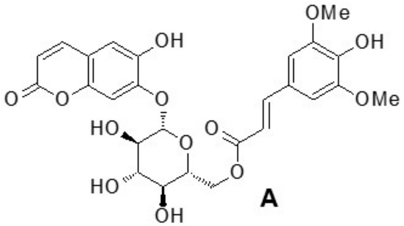

A

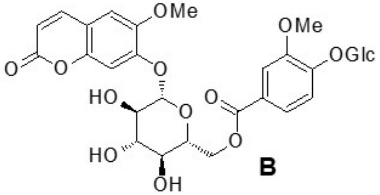

B

Position	3	A	B*
solvent	Pyridine-d_5	MeOD	DMSO-d_5
2	161.6	163.5	160.7
3	114.5	112.8	113.3
4	144.0	145.9	144.3
4a	113.5	112.7	112.4
5	110.2	116.4	110.1
6	147.4	144.2	145.9
7	151.5	153.3	149.7
8	104.6	104.6	102.8
8a	150.8	152.5	148.9
6-OCH_3	56.5	-	56.0
1′	102.3	103.6	99.2
2′	74.9	74.8	72.8
3′	78.7	77.4	76.4
4′	71.6	71.9	70.0
5′	76.1	75.9	73.1
6′	64.6	64.8	64.5
1″	127.1	126.5	122.6
2″	116.5	107.1	112.5
3″	147.8	149.5	148.5
4″	149.8	139.9	150.5
5″	117.3	149.5	113.9
6″	122.5	107.1	123.1
7″	146.6	147.4	165.3
8″	114.9	115.8	
9″	167.9	168.7	
3″,5″-OMe		56.9	55.5

* One of the glucose signals was omitted.

Position	3	A	B*
solvent	pyridine-d_5	MeOD	DMSO
3	6.32 (*d*, 9.5)	5.79 (*d*, 9.3)	6.32 (*d*, 9.5)
4	7.65 (*d*, 9.5)	7.60 (*d*, 9.3)	7.99 (*d*, 9.5)
5	7.01 (*s*)	7.27 (*s*)	7.31 (*s*)
8	7.568 (*s*)	6.77 (*s*)	7.27 (*s*)
6-OCH_3	3.69 (3H, *s*)	-	3.82 (3H, *s*)
1'	5.80 (*d*, 7.3)	4.86 (*d*, 7.2)	5.24 (*d*, 7.4)
2'	4.42 (*dd*-like, 8.8, 7.3)	-	3.36 (*m*)
3'	4.44 (br *t*, 8.8)	-	3.38 (*m*)
4'	4.24 (br *t*, 8.8)	-	3.25 (*dd*, 9.1, 5.2)
5'	4.37 (*ddd*, 9.5, 6.5, 1.9)	-	3.95 (*ddd*, 9.1, 8.0, 1.6)
6'	4.91 (*dd*, 11.9, 6.5)	4.60 (*m*)	4.66 (*d*, 11.8)
	5.11 (*dd*, 11.9, 1.9)	-	4.08 (*dd*, 11.8, 8.0)
2''	7.570 (*d*, 1.8)	6.88 (*s*)	7.44 (*d*, 2.0)
5''	7.31 (*d*, 8.3)	-	7.19 (*d*, 8.4)
6''	7.27 (*dd*, 8.3, 1.8)	6.88 (*s*)	7.57 (*dd*, 8.4, 2.0)
7''	7.92 (*d*, 15.9)	7.61 (*d*, 15.6)	-
8''	6.69 (*d*, 15.9)	6.43 (*d*, 15.6)	-
3'',5''-OMe		3.87 (3H, *s*)	3.77 (3H, *s*)

* One of the glucose signals was omitted.

A: 6′-*O*-sinapinoyl esculin

B: Indidene F

Table S2: Reported IC_{50} values of the identified known compounds.

Anti-*Trypanosoma* activity			
	**IC_{50} (µg/ml)**	**strains**	**Ref**
**6**	17.0 ± 0.14	*Trypanosoma brucei brucei* TC221	16
**7**	398	*Trypanosoma cruzi* (trypomastigote)	17
	127.7	*Trypanosoma cruzi* (trypomastigote)	18
**8**	6.0 ± 0.13	*Trypanosoma congolense*	19
**9**	29.692 ± 0.575	*Trypanosoma brucei brucei*	20
**18**	7.7	*Trypanosoma cruzi* (trypomastigote and amastigote) Tulahuen strain	21

Anti-*Plasmodium* activity			
	**IC_{50} (µg/ml)**	**strains**	
**6**	11.52	*Plasmodium falciparum*	22
**7**	3.1 ± 0.01	*Plasmodium falciparum* FCR-3 strain (Chloroquine-sensitive)	23
**8**	7.15 ± 0.006	*Plasmodium falciparum* FCR-3 strain (Chloroquine-sensitive)	23
**9**	10.1 ± 1.3	*Plasmodium falciparum* D10 strain (Chloroquine-sensitive)	24
	6.1 ± 3.8	*Plasmodium falciparum* W2 strain (Chloroquine-resistant)	24
**12**	2.39	*Plasmodium falciparum* K1 strain (multidrug-resistant)	25
**13**	2.9	*Plasmodium falciparum* K1 strain (multidrug-resistant)	25
**15**	1.84	*Plasmodium falciparum* K1 strain (multidrug-resistant)	26
	18.4	*Plasmodium falciparum* 3D7 strain (Chloroquine-sensitive)	26
**18**	83.4 ± 10.0	*Plasmodium falciparum* Dd2 strain (Chloroquine-resistant)	27
